# Genome-Wide Association Mapping in a Rice MAGIC Plus Population Detects QTLs and Genes Useful for Biofortification

**DOI:** 10.3389/fpls.2018.01347

**Published:** 2018-09-20

**Authors:** Gwen Iris L. Descalsota, B. P. Mallikarjuna Swamy, Hein Zaw, Mary Ann Inabangan-Asilo, Amery Amparado, Ramil Mauleon, Prabhjit Chadha-Mohanty, Emily C. Arocena, Chitra Raghavan, Hei Leung, Jose E. Hernandez, Antonio B. Lalusin, Merlyn S. Mendioro, Ma. Genaleen Q. Diaz, Russell Reinke

**Affiliations:** ^1^Strategic Innovation Platform, International Rice Research Institute, Manila, Philippines; ^2^University of Southern Mindanao, Kabacan, Philippines; ^3^Philippine Rice Research Institute, Science City of Muñoz, Philippines; ^4^University of the Philippines Los Baños, Los Baños, Philippines

**Keywords:** biofortification, Fe, Zn, GWAS, rice, *Xanthomonas oryzae*, MAGIC, Bayesian network

## Abstract

The development of rice genotypes with micronutrient-dense grains and disease resistance is one of the major priorities in rice improvement programs. We conducted Genome-wide association studies (GWAS) using a Multi-parent Advanced Generation Inter-Cross (MAGIC) Plus population to identify QTLs and SNP markers that could potentially be integrated in biofortification and disease resistance breeding. We evaluated 144 MAGIC Plus lines for agronomic and biofortification traits over two locations for two seasons, while disease resistance was screened for one season in the screen house. X-ray fluorescence technology was used to measure grain Fe and Zn concentrations. Genotyping was carried out by genotype by sequencing and a total of 14,242 SNP markers were used in the association analysis. We used Mixed linear model (MLM) with kinship and detected 57 significant genomic regions with a -log10 (*P*-value) ≥ 3.0. The *PH*_1.1_ and *Zn*_7.1_ were consistently identified in all the four environments, ten QTLs *qDF*_3.1_, *qDF*_6.2_ *qDF*_9.1_ *qPH*_5.1_ *qGL*_3.1_, *qGW*_3.1_, *qGW*_11.1_, and *qZn*_6.2_ were detected in two environments, while two major loci *qBLB*_11.1_ and *qBLB*_5.1_ were identified for Bacterial Leaf Blight (BLB) resistance. The associated SNP markers were found to co-locate with known major genes and QTLs such as *OsMADS50* for days to flowering, *osGA20ox2* for plant height, and *GS3* for grain length. Similarly, *Xa4* and *xa5* genes were identified for BLB resistance and *Pi5(t), Pi28(t)*, and *Pi30(t)* genes were identified for Blast resistance. A number of metal homeostasis genes *OsMTP6*, *OsNAS3*, *OsMT2D*, *OsVIT1*, and *OsNRAMP7* were co-located with QTLs for Fe and Zn. The marker-trait relationships from Bayesian network analysis showed consistency with the results of GWAS. A number of promising candidate genes reported in our study can be further validated. We identified several QTLs/genes pyramided lines with high grain Zn and acceptable yield potential, which are a good resource for further evaluation to release as varieties as well as for use in breeding programs.

## Introduction

Micronutrient deficiencies and their associated health risks have become a major global health burden. An estimated two billion people, particularly children and pregnant and lactating women, suffer micronutrient malnutrition. Recently, the United Nations (UN) declared that tackling micronutrient deficiencies is one of the sustainable development goals to be achieved by 2035. Various agencies have made significant efforts on a global scale to combat micronutrient deficiencies through preventive supplementation, fortification, and biofortification approaches (Dalmiya and Schultink, 2003). However, biofortification of major staple crops is considered as the most cost-effective and sustainable approach to tackle hidden hunger. The successful effort by HarvestPlus to biofortify sweet potato with vitamin A targeted to Africa and Latin America was awarded the World Food Prize for the year 2016. Recognition of this significant feat has provided an added impetus to expedite the development and release of biofortified staple crops targeted to different regions of the world to make a global impact on health and nutrition.

Among different micronutrients, iron (Fe) and zinc (Zn) are the most essential for human health and nutrition. Zn is a major co-factor for several vital enzymes involved in metabolic activities (Frassinetti et al., 2006; Roohani et al., 2013; Sadeghzadeh, 2013), whereas Fe is an essential component of hemoglobin and myoglobin. Zn deficiency causes stunting, diarrhea, loss of appetite, and impaired immune function (Prasad, 2003; Hotz and Brown, 2004; Wang and Busbey, 2005), while Fe deficiency is highly associated with anemia. These deficiencies are strongly linked to diets in which energy is primarily derived from the consumption of cereals, which are a poor source of minerals (Shahzad et al., 2014). Therefore, there is significant potential and opportunity to develop and release biofortified rice varieties targeted to poor and marginalized rice-consuming Asian populations to overcome malnutrition (Hefferon, 2015).

Rice is the major staple food and source of energy for more than half of the world’s population, but the currently grown popular high-yielding rice varieties are a poor source of essential micronutrients in their polished (white) form (Kennedy and Burlingame, 2003; Sharma et al., 2013). Within cultivated rice germplasm, there is enough genetic variability for grain Zn in the polished rice but not for grain Fe (Swamy et al., 2016). Hence, it is possible to breed for high-Zn rice through breeding approaches, while a transgenic approach is feasible for the development of high-Fe rice (Trijatmiko et al., 2016). Several high-Zn rice varieties have been successfully developed and released for cultivation by conventional breeding approaches, but this process is slow due to the complex genetic nature of high grain Zn, the lack of field-based phenotyping techniques and tightly linked markers, and significant genotype and environmental interactions (Gande et al., 2014; Zhang et al., 2014). The recent advances in molecular technologies, rice genome sequences, and genome-wide markers have enabled the genetic dissection of complex traits to identify major-effect quantitative trait loci (QTLs) and genes. These loci, in turn, can be transferred to different genetic backgrounds more precisely through marker-assisted breeding and genomics-assisted selection approaches leading to the faster development of rice varieties (Collard and Mackill, 2008; Swamy and Kumar, 2012).

A successful biofortified product should be high yielding with good grain quality traits, resistant to major pests and diseases prevailing in the target environments, in addition to high grain mineral concentration (Bouis, 2003; Welch and Graham, 2004). An understanding of the molecular basis of all of these complex traits will help in precisely pyramiding several genes and QTLs to develop superior and farmer-adoptive rice varieties. Biparental mapping and genome-wide association studies (GWAS) have been carried out to map QTLs/genes for various traits in rice (Collard and Mackill, 2008; Norton et al., 2014; Zhang et al., 2014; Nawaz et al., 2015). GWAS have several advantages over biparental mapping such as high mapping resolution and the identification of multiple and rare alleles (Nordborg and Tavaré, 2002). GWAS was able to identify QTLs for grain Fe, Zn, and several other mineral elements (Norton et al., 2010; Zhang et al., 2014). In addition, 31 putative QTLs were identified for Fe, Zn, Mn, Cu, Ca, Mg, P, and K contents with introgression lines derived from a cross between elite *indica* cultivar Teqing and the wild rice *Oryza rufipogon* (Garcia-Oliveira et al., 2009). Moreover, 14 QTLs for Fe and Zn as well as high-priority candidate genes were identified such as *OsYSL1* and *OsMTP1* for Fe and *OsARD2, OsIRT1, OsNAS1*, and *OsNAS2* for Zn in rice seeds (Anuradha et al., 2012). GWAS approach has also been used to identify major loci for different elemental concentrations such as Ca, Fe, and Zn in wheat (Velu et al., 2016; Alomari et al., 2017), Fe and Zn in barley (Mamo et al., 2014; Gyawali et al., 2017), and Fe and Zn in maize (Hindu et al., 2018).

In recent years, Multi-parent Advanced Generation Inter-cross (MAGIC) populations have become popular genetic resources for mapping and for developing breeding lines with multiple desirable traits (Kover et al., 2009; Bandillo et al., 2013; Meng et al., 2016). MAGIC populations have a relatively wide genetic background without significant population structure, which is a major constraint in association mapping using diversity panels. The further-refined MAGIC Plus population with additional generations of intermating has increased levels of recombination and thus greater mapping resolution (Bandillo et al., 2013). Thus, MAGIC Plus rice lines can be a powerful genetic resources to facilitate QTL analyses/gene discovery with high mapping resolution for both simple and complex traits.

Our study reports on the mapping of loci for disease resistance, grain micronutrients (Fe and Zn), yield, and yield-related traits using the MAGIC Plus population developed at IRRI. The main objectives were to (a) assess the utility of the MAGIC Plus population for QTL mapping of important agronomic, biofortification, and disease resistance traits; (b) identify MAGIC Plus lines with a combination of good agronomic traits and high grain Zn, (c) identify QTLs and SNP markers that could potentially be integrated in biofortification and disease resistance breeding, and (d) identify candidate genes associated with major effect QTLs for agronomic, biofortification, and disease resistance traits.

## Materials and Methods

### Materials

The MAGIC population was developed at IRRI by inter-crossing eight elite *indica* founder lines that possessed good grain quality, high yield potential and biotic and abiotic stress tolerance (Bandillo et al., 2013). The selected MAGIC lines underwent two additional rounds of 8-way F_1_ inter-crossing to produce the MAGIC Plus population (Bandillo et al., 2013). The MAGIC Plus lines were evaluated during the 2015 dry season (DS) and 2015 wet season (WS) in two locations: the Robert Zeigler Experiment Station at the International Rice Research Institute (IRRI), Los Baños (LB), Laguna, and the Philippine Rice Research Institute (PhilRice), Muñoz (MU), Nueva Ecija. All 144 entries were included for testing in LB, while only 142 entries were included in MU. The four different environments were designated as E1 (LB-DS), E2 (LB-WS), E3 (MU-DS), and E4 (MU-WS).

### Phenotyping

All trials were laid out in a randomized complete block design (RCBD) with three replications in LB and two replications in MU. We transplanted seedlings that were 21 days old with a uniform spacing of 20 cm ×20 cm. Standard agronomic practices and plant protection measures were applied to ensure good crop growth and complete grain development. All lines were phenotyped for agronomic and biofortification traits such as days to 50% flowering (DF), plant height (PH), number of tillers (NT), number of panicles (NP), panicle length (PL), grain length (GL), grain width (GW), number of filled grains per panicle (FG), thousand-grain weight (TGW), yield per hectare (YLD), grain iron concentration (Fe), and grain zinc concentration (Zn) in E1 and E2. On the other hand, only five agronomic and biofortification traits (PH, NP, YLD, Fe, and Zn) were evaluated in E3 and E4. All the traits were measured following the standard evaluation system (IRRI, 2013). Fe and Zn were measured using XRF-Bruker S2 Ranger. We used 3 g of milled rice samples for measuring grain Fe and Zn concentration. The samples were analyzed twice and expressed in parts per million (ppm). The average Fe and Zn values were used for the statistical analysis. All the basic statistical parameters and correlations were generated using STAR v.2.0.1. Analysis of variance (ANOVA) was carried out using PBTools v1.4.

### Genetic Analysis

SNP marker data were generated by genotype-by-sequencing (GBS) and screened based on ≥90% call rate, locus homozygosity, and minor allele frequency (MAF) ≥0.05. Population structure was determined by setting the number of groups (K) from 1 to 10 and at each K structure was analyzed with six replications. Each run was implemented with a burn-in period of 10,000 steps followed by 10,000 Monte Carlo Markov Chain iterations. The optimal number of K clusters was estimated with the parameter (ΔK) (Evanno et al., 2005) in Structure Harvester (Earl and VonHoldt, 2012). The intra-chromosomal LD (*r*^2^-values) between SNP marker pairs was calculated using TASSEL v5.2.20. Marker pairs with statistically significant LD (*p* < 0.05) were considered in the LD analysis. The r^2^ values were plotted against distance (Mb) and a smooth line was fitted using R software package ggplot2 (Wickham, 2009).

### Statistical Analysis

#### Association Mapping

A total of 14,242 SNP markers were used in the genome-wide association study (GWAS). The average trait values of each line in the MAGIC Plus population were used for association analysis. We used Trait Analysis by association, Evolution and Linkage (TASSEL5.2.2) package for the association analysis (Bradbury et al., 2007). The mixed linear model (MLM) with Kinship approach in TASSEL v.5.2.20 was used to carry out GWAS. Comparisons between models with and without structure as covariate did not differ significantly. Therefore, the structure covariate was not included in the final analyses. Manhattan plots were produced and a threshold value for declaring marker-trait association was set at –log (*p*-value) ≥ 3.0 (i.e., *p*-value ≤ 0.001). QQ plots for each trait were examined to determine whether the model could control false positives, which, in turn, indicate the suitability of the model for analysis. We also carried out Benjamini-Hochberg method in ‘multtest’ package in R to adjust *p*-values from TASSEL to detect false positives. QQ plots that showed SNP markers rising above the line toward the tail of the distribution indicate that positions of strong causal polymorphisms were detected.

#### Bayesian Network Analysis

The number of SNP markers was further reduced from 14,242 to 7,634 by eliminating those that are in high LD with adjacent markers. Missing calls of the 7,634 evenly distributed SNP markers were imputed using Beagle v4. BN analysis was carried out using best linear unbiased predictions (BLUPs) data from all environments, and SNP markers through the packages R/lme4, R/bnlearn, and R/parallel. The functions of each package were to adjust family structure, learn the model and perform predictions, and hasten learning, respectively. The type I error α was set at 0.01.

### Candidate Gene Analysis for Biofortification and New Blast Resistance QTLs

The search for candidate genes from previous studies was accomplished using the Rice Annotation Project (RAP) database genome browser^1^. The physical positions of genes related to traits of interest were determined and those that were physically located near identified QTLs were considered candidate genes. Further searches using the Gramene database^2^ were performed for previously reported QTLs that co-localized with present QTLs for agronomic traits.

Novel candidate genes from two new *indica* reference genomes (IR8 and Minghui 63) were identified by (1) lifting-over the physical coordinates of QTLs identified by GWAS from Nipponbare to the two references and (2) identifying the predicted genes underlying the lifted-over QTLs. QTL boundaries were determined by aligning the +/- 60 bases flanking the QTL boundaries defined from GWAS to the two *indica* references. The definitive alignments were selected based on strict criteria (at least 120 bp aligned at maximum one mismatch), and lifted-over QTLs were selected based on being located on the same chromosome as Nipponbare with a size of <1 M bps. Alignment between genes within QTLs of the two reference genomes against Nipponbare genes (RGAP 7.0) (Kawahara et al., 2013) was carried out using BLASTN (v 2.4.0), and equivalent genes were identified based on alignment of at least 90% of the gene length with > = 95% identity. IR8 and Minghui 63 genes that did not align to Nipponbare were further characterized by aligning to the NR/NT database at NCBI (March 6, 2017 release).

All the SNPs in 3K genome panel were used for filtering the SNPs in the selected genomic regions between IR69428-6-1-1-3-3 and IR64 using Rice SNP-Seek Database^3^. We used 1kb upstream region of the each gene for short listing the SNPs in the promoter regions. All the polymorphic SNPs both in the promoter and coding regions of the selected genes were shortlisted.

### Gene Expression Analysis

#### RNA Isolation and cDNA Synthesis

Five biological replicates per genotype (low Zn IR64, high Zn IR69428, and high Zn BR7840) were collected at two time points: 11 and 15 days after flowering (DAF). IR64 was used as the check variety. Panicles were immediately frozen in liquid nitrogen to preserve RNA integrity. Total RNA was isolated from grains using PureLink Plant RNA Reagent (Life Technologies, CA, United States) and purified using RNeasy Mini Kit with on-column DNAse treatment using RNase-Free DNase Set (Qiagen, CA, United States) as described by the manufacturers. RNA was eluted in 30 μl DEPC-treated water, quantified using a NanoDrop 1000 spectrophotometer (Thermo Fisher Scientific, DE, United States), and integrity-checked on agarose gel. On a 96-well plate, ∼1 μg RNA, 2 μl 10× loading dye, and DEPC-treated water (total volume of 20 μl) were mixed and denatured at 70°C for 1 min and then immediately placed on ice for 10 min prior to running in 1% agarose gel at 180V for 45 min.

Reverse transcription was done with 1 μg total RNA, 1 μl Oligo DT, 1 μl anchored-oligo primer from Transcriptor First Strand cDNA Synthesis Kit (Roche Applied Science, Mannheim, Germany), and PCR-grade water to make a total volume of 13 μl. Tubes were incubated at 65°C for 10 min and then placed on ice. The following components were added to the tubes in order: 4 μl Transcriptor Reverse Transcriptase Reaction Buffer, 0.5 μl Protector RNase Inhibitor, 2 μl Deoxynucleotide Mix, and 0.5 μl Transcriptor Reverse Transcriptase. The tubes were spun down and placed in the thermal cycler at 55°C for 30 min and 85°C for 5 min. NRT controls were also included and cDNA was diluted to 1:10.

#### Primer Design and Real-time qRT-PCR Analysis

Transcript sequences of three reference and seven target genes were retrieved from RAP-DB. Primers were designed using Primer3Plus on the 3’ end with 70–200 bp amplicon size and Tm between 60 and 62°C. Hairpins, dimers prediction, and amplification specificity were checked using MFEprimer 2.0 and by blast on RAP-DB.

Real-time qRT-PCR was performed on LightCycler 480 Instrument II (Roche) in 40 cycles using SYBR Select Master Mix (Applied Biosystems, CA, United States) for 10 μl reaction with 2 μl cDNA in duplicate. Assays were performed following a sample maximization format, with NTC and IRC. Five biological replicates were used for the analysis, each replicate constituted from pooled seeds of five panicles or of a pool of five flag leaves per plant; samples were frozen in liquid nitrogen. Tissues were ground and total RNA extracted to prepare five separate pools for each tissue. Efficiency of primers was determined using 5- or 2-fold dilution series of cDNA pool. Acceptable efficiency was set between 1.8 and 2.1.

Normalization of gene expression was performed as previously described^1^ using the geometric mean of three reference genes (*OsTPI*, *OsUBC32*, and *OsTCTP*). Cp values of reactions with no or low signal were set to 35 and technical replications were averaged. Amplicon similarity between the genotypes was tested by Tm comparison. Sample clustering was done using the Euclidian distance of normalized relative quantity (NRQ) values and statistics using analysis of variance and Tukey’s HSD at *p*-value ≤ 0.05.

## Results

### Phenotypic Analysis of the MAGIC Plus Population

**FIGURE 1:**
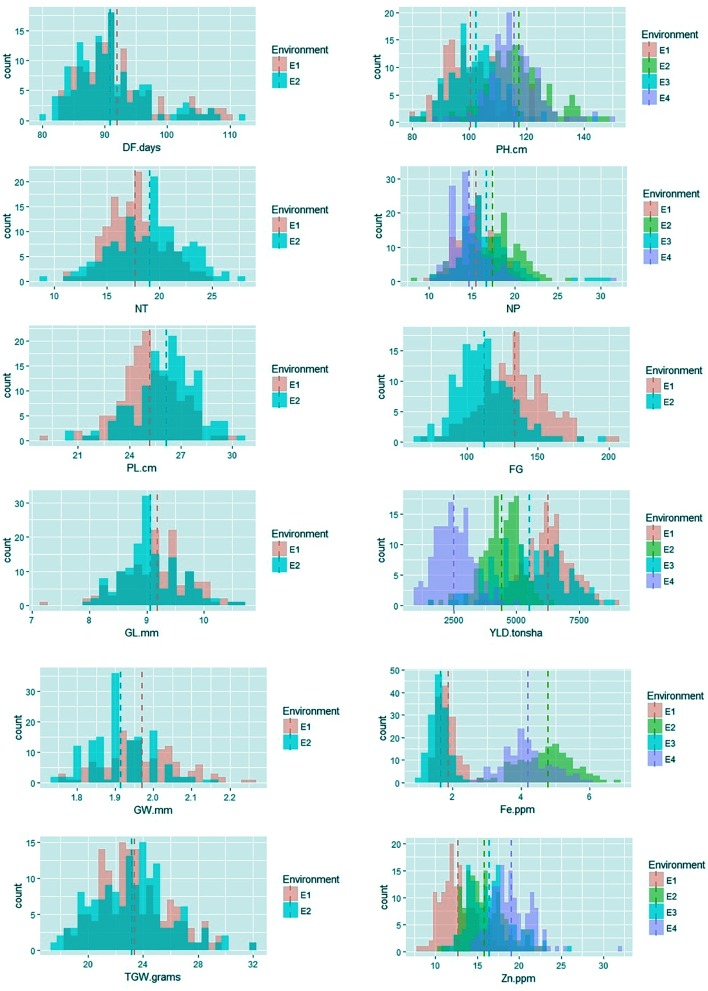
Frequency distribution of agronomic and biofortification traits in the MAGIC Plus population evaluated in four environments. DF, days to 50% flowering; PH, plant height (cm); NT, number of tillers; NP, number of panicles; PL, panicle length (cm); GL, grain length (mm); GW, grain width (mm); FG, number of filled grains; TGW, thousand grain weight (g); YLD, grain yield (Kg/ha); Fe, iron (ppm); Zn, zinc (ppm); E, denotes environments in which MAGIC Plus population was evaluated; E1: IRRI, Los Baňos, Laguna during 2015DS; E2: IRRI, Los Baňos, Laguna during 2015WS; E3: PhilRice, Muňoz, Nueva Ecija during 2015DS; E4: PhilRice, Muňoz, Nueva Ecija during 2015WS; Dashed bars indicate mean of the population in each environment; The Y-axis shows the frequency and the X-axis shows the distribution for different traits, Different color codes indicates environments.

Among the traits evaluated, a majority of the agronomic traits (PH, NT, PL, FG, TGW, and YLD) showed normal distributions in all environments indicating their quantitative nature (**Figure 1**). The range, mean, standard deviation, coefficient of variation (CV), and heritability values for different traits in different environments are provided in **Table 1**. Significant phenotypic variation was observed for all traits. The highest values for the agronomic traits PH (150 cm) and NP (31) were recorded in E4 while the highest YLD (8945 kg/ha) was recorded in E1. The highest values for biofortification traits Fe (6.0 ppm) and Zn (32.0 ppm) were recorded in E4. The CV was low (<10%) for most agronomic traits (DF, PH, PL, GL, and GW) whereas moderate CV (<30%) was reported for other traits. The contribution of genotypic variance to the total phenotypic variance was significant for almost all traits. The broad-sense heritability for all the traits ranged from 18 to 98%. High heritability (>70%) was observed for the majority of the agronomic traits (DF, PH, PL, GL, and TGW) and the Zn biofortification trait.

**Table 1:** Summary of statistics for agronomic and biofortification traits in the MAGIC Plus population.

Trait	Range	Mean ± SE	*F*-value	CV (%)	H^2^	Environment
DF	82.7–109.7	91.96 ± 0.53	31.12^∗∗^	6.9	0.97	E1
	80.3–112.5	90.89 ± 0.51	54.97^∗∗^	6.7	0.98	E2
PH (cm)	79.7–134.1	100.49 ± 0.83	9.00^∗∗^	9.9	0.89	E1
	83.9–148.9	117.29 ± 0.92	10.46^∗∗^	9.4	0.89	E2
	80.4–143.1	102.25 ± 0.78	2.75^∗∗^	9.2	0.97	E3
	87.1–150.0	115.66 ± 0.79	4.20^∗∗^	8.1	0.76	E4
NT	11.3–24.9	17.69 ± 0.22	1.89^∗∗^	14.8	0.47	E1
	9.0–28.0	19.05 ± 0.29	2.30^∗∗^	18.0	0.51	E2
NP	10.0–21.0	15.48 ± 0.19	1.62^∗∗^	14.4	0.38	E1
	8.1–26.2	17.41 ± 0.26	2.19^∗∗^	18.0	0.55	E2
	10.3–30.5	16.70 ± 0.28	1.37^∗∗^	20.2	0.85	E3
	10.8–31.6	14.67 ± 0.20	0.99	16.3	0.19	E4
PL (cm)	19.0–30.1	25.18 ± 0.14	4.91^∗∗^	6.8	0.79	E1
	20.4–30.4	26.14 ± 0.15	3.82^∗∗^	7.1	0.74	E2
GL (mm)	7.2–10.6	9.18 ± 0.05	11.50^∗∗^	6.2	0.91	E1
	7.9–10.7	9.07 ± 0.04	22.01^∗∗^	5.6	0.95	E2
GW (mm)	1.8–2.3	1.97 ± 0.01	2.47^∗∗^	5.2	0.59	E1
	1.7–2.2	1.92 ± 0.01	5.41^∗∗^	4.5	0.81	E2
FG	71.7–205.8	133.77 ± 1.94	2.75^∗∗^	17.4	0.64	E1
	64.8–193.3	112.65 ± 1.70	2.10^∗∗^	18.1	0.48	E2
TGW (g)	18.5–32.1	23.36 ± 0.22	10.74^∗∗^	11.2	0.91	E1
	17.3–31.8	23.18 ± 0.23	9.24^∗∗^	12.2	0.89	E2
YLD (Kg/ha)	3667.2–8944.6	6270.24 ± 78.95	3.44^∗∗^	15.1	0.71	E1
	1659.5–6366.5	4420.84 ± 64.53	3.30^∗∗^	17.5	0.70	E2
	1699.4–8732.2	5518.24 ± 118.76	0.75	25.1	0.55	E3
	935.7–4954.9	2506.98 ± 63.32	2.32^∗∗^	30.1	0.57	E4
Fe (ppm)	1.4–2.9	1.89 ± 0.02	2.08^∗∗^	15.4	0.52	E1
	2.8–6.9	4.78 ± 0.06	1.97^∗∗^	16.0	0.49	E2
	1.0–2.4	1.66 ± 0.02	0.22	14.7	0.32	E3
	2.7–6.0	4.20 ± 0.06	1.21	21.8	0.18	E4
Zn (ppm)	8.1–20.3	12.73 ± 0.18	4.18^∗∗^	16.6	0.76	E1
	10.7–22.8	15.82 ± 0.20	4.83^∗∗^	15.0	0.79	E2
	11.0–26.2	16.47 ± 0.21	4.72^∗∗^	15.4	0.79	E3
	14.4–32.0	19.10 ± 0.22	6.57^∗∗^	14.4	0.85	E4


*DF, days to 50% flowering; PH, plant height (cm); NT, number of tillers; NP, number of panicles; PL, panicle length (cm); GL, grain length (mm); GW, grain width (mm); FG, number of filled grains; TGW, thousand grain weight (g); YLD, grain yield (Kg/ha); Fe, iron (ppm); Zn, zinc (ppm); CV, co-efficient of variation (%); H^2^, heritability; SE, standard error; ^∗∗^Significant at P ≤ 0.01.*

**FIGURE 2:**
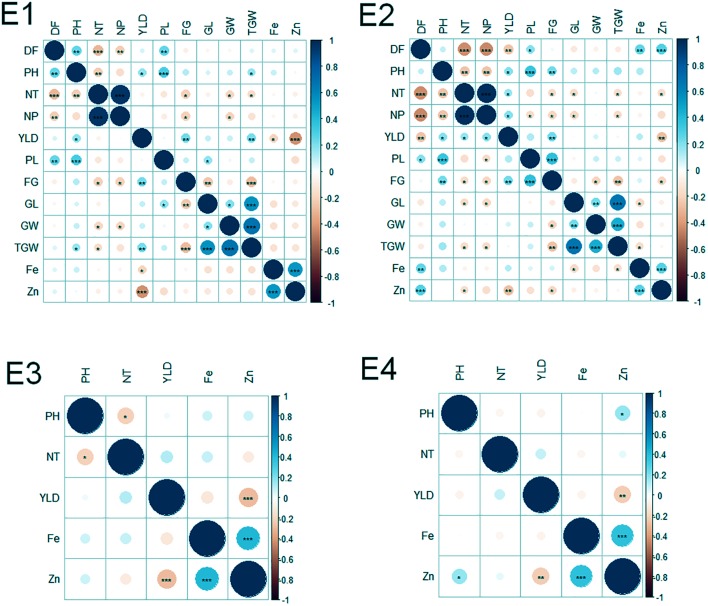
Correlations among agronomic and biofortification traits in the MAGIC Plus population evaluated in four environments. DF, days to 50% flowering; PH, plant height (cm); NT, number of tillers; NP, number of panicles; PL, panicle length (cm); GL, grain length (mm); GW, grain width (mm); FG, number of filled grains; TGW, thousand grain weight (g); YLD, grain yield (Kg/ha); Fe, iron (ppm); Zn, zinc (ppm); E, denotes environments in which MAGIC Plus population was evaluated; E1: IRRI, Los Baňos, Laguna during 2015DS; E2: IRRI, Los Baňos, Laguna during 2015WS; E3: PhilRice, Muňoz, Nueva Ecija during 2015DS; E4: PhilRice, Muňoz, Nueva Ecija during 2015WS; The test of significance were indicated as ^∗∗∗^*p* < 0.001, ^∗∗^*p* < 0.01, ^∗^*p* < 0.05. The blue color shows the positive association and red color indicates the negative association and the intensity of the color indicates degree of association.

Of the 152 possible correlations among agronomic and biofortification traits in four environments, 65 correlations were significant (**Figure 2**). The number of significant correlations was higher in the WS (E2 and E4) than in the DS (E1 and E3). Notable significant correlations were found among agronomic traits (DF and NT; NT and NP; PH and PL; GL and TGW; GW, and TGW; and FG and YLD), between agronomic and biofortification traits (YLD and Zn), and among biofortification traits (Zn and Fe).

For the disease resistance traits, the MAGIC Plus lines and eight parents were screened against four races of bacterial leaf blight (BLB) pathogens (*PXO61*, *PXO86*, *PXO99*, and *PXO341*) and one race of blast pathogen. Six parents were resistant to *PXO61*, one parent was resistant to *PXO86*, four parents were resistant to *PXO99*, seven parents were resistant to *PXO341*, and six parents were resistant to blast pathogen. In the screening of four races of BLB, a total of 117 lines out of 144 MAGIC Plus lines showed resistance against *PXO61*, 10 were resistant to *PXO86*, 49 lines showed resistance against *PXO99*, and 114 lines showed resistance against *PXO341*. For blast resistance, 114 lines out of 144 lines were resistant to natural blast infection. Out of 144 MAGIC Plus lines, the 9 best lines were identified to possess good yield potential, biofortification, and disease resistance characteristics (**Table 2**). The combinations of favorable traits in these lines make them highly suitable for direct use in breeding.

**Table 2:** MAGIC Plus lines with high grain Zn, high grain yield and disease resistance.

Designation	2015 DS			2015 WS			Screen house/blast nursery				
			
	Zn	Fe	YLD Kg/ha	Zn	Fe	YLD Kg/ha	PXO61	PXO86	PXO99	PXO341	Blast
									
	ppm			ppm							
IR95028:3-B-3-7-20-GBS	15.9	1.8	5361	19.6	4.8	3362	R	MR	R	R	MR
IR95044:3-B-17-10-17-GBS	16.3	1.9	5888	18.8	4.6	3616	R	MR	R	R	R
IR95044:4-B-7-21-21-GBS	15.5	1.9	5686	19.1	4.6	3953	R	MR	R	R	R
IR95044:5-B-12-5-6-GBS	16.3	1.8	5068	18.7	4.3	3481	R	MR	R	R	R
IR95044:17-B-6-18-22-GBS	15.8	1.8	6614	18.1	4.4	4021	R	MS	R	R	R
IR95058:6-B-6-21-10-GBS	16.9	1.8	5689	19.1	4.6	3129	R	MR	MS	R	MR
IR95095:3-B-11-19-8-GBS	18.3	1.8	5778	19.2	4.7	3530	R	MR	MR	R	R
IR95132:3-B-18-13-11-GBS	18.1	1.9	5638	20.2	4.7	3314	R	MS	MR	R	MR
IR95132:4-B-3-10-19-GBS	16.2	1.9	5680	19.4	4.6	3288	R	R	R	R	R


*YLD, grain yield (Kg/ha); Fe, iron (ppm); Zn, zinc (ppm); R, resistant; MR, moderately resistant; MS, moderately susceptible.*

### Genetic Analysis of the MAGIC Plus Population

**FIGURE 3:**
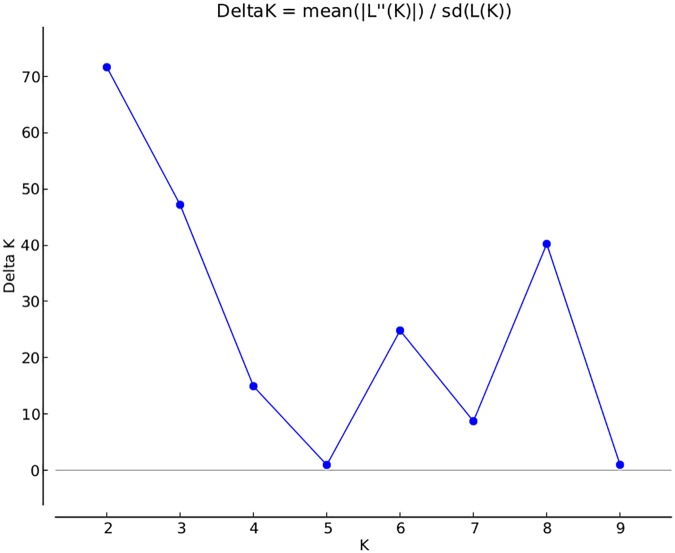
Population structure in the MAGIC Plus population. The X-axis shows the number of subgroups (K) and Y-axis shows rate change of log probability values (ΔK) with change in K. The highest ΔK is 70 at *K* = 2.

**FIGURE 4:**
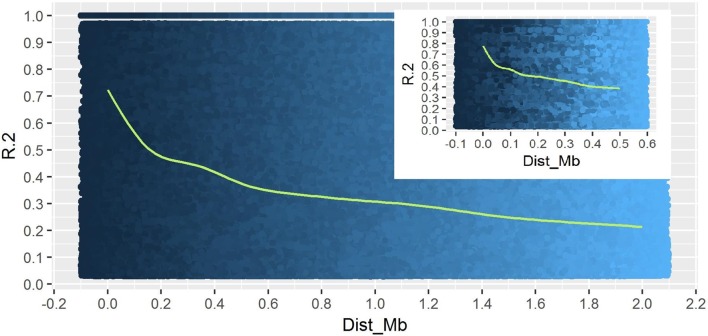
Linkage Disequilibrium decay plot of MAGIC Plus population. The X-axis represents physical distance in mega bases (Mb) and the Y-axis represents chromosomal co-efficient of determination (*r*^2^).

A total of 14,242 SNP markers, with an average density of 30.1 kb, were used for population structure analysis. The results revealed that the variance of log likelihood increased from K = 1 to K = 10 and the highest ΔK of only 70.3 was observed at K = 2 (**Figure 3**), indicating that there was insignificant population structure. Association analyses with kinship, and with and without cluster membership of each individual were carried out and results were compared. Linkage disequilibrium (LD) analysis carried out in the MAGIC Plus population showed that only 2,157,869 (21.29%) out of the 9,843,868 total marker pairs had significant (*p* < 0.05) LD. Variations in the r^2^ values of significant intra-chromosomal marker pairs across different physical distance groups were observed (**Table 3**). The average LD was high (*r*^2^ = 0.796) at short distances (<5 kb) and declined to 50% around 400 kb. The decay was steady but still showed substantial residual LD (*r*^2^ > 0.2) at a distance of 2.0 Mb. A high percentage of marker pairs in complete LD can be observed in the short-distance group while marker pairs in complete LD were negligible in the long-distance group (>7.5 Mb). Scatter plots of intra-chromosomal r^2^ against physical distance show a clean pattern of LD decay (**Figure 4**).

**Table 3:** Linkage disequilibrium in the MAGIC Plus population.

Distance (kb)	*r*^2^ (*p* < 0.05)	Significant LD pairs	Marker pairs in complete LD	Marker pairs in complete LD (%)
0–5	0.796	12,440	8,153	65.54
>5–100	0.619	65,140	23,507	36.09
>100–250	0.506	84,254	15,808	18.76
>250–500	0.421	120,362	12,390	10.29
>500–750	0.350	108,080	6,011	5.56
>750–1000	0.318	95,907	3,138	3.27
>1000–1500	0.280	164,489	4,970	3.02
>1500–2000	0.230	141,971	2,601	1.83
>2000–2500	0.200	115,415	1,488	1.29
>2500–5000	0.167	393,990	3,476	0.88
>5000–7500	0.133	232,003	233	0.10
>7500	0.075	623,818	7	0.00
**Total**		**2,157,869**	**81,782**	


*LD, linkage disequilibrium; kb, kilo bases; r^2^, co-efficient of determination.*

### GWAS Identified Common and Environment-Specific QTLs, and Candidate Genes

We carried out separate association analysis for each environment and also using BLUPs for all the four environments (results not shown). Most of the major effect loci identified in individual environments except for two loci one each for NT and Fe concentration were also detected in BLUPs. Since G x E is an important aspect of biofortification, we identified loci that were specific to individual environments and the ones which were consistently expressed across the locations.

#### Agronomic Traits

**FIGURE 5:**
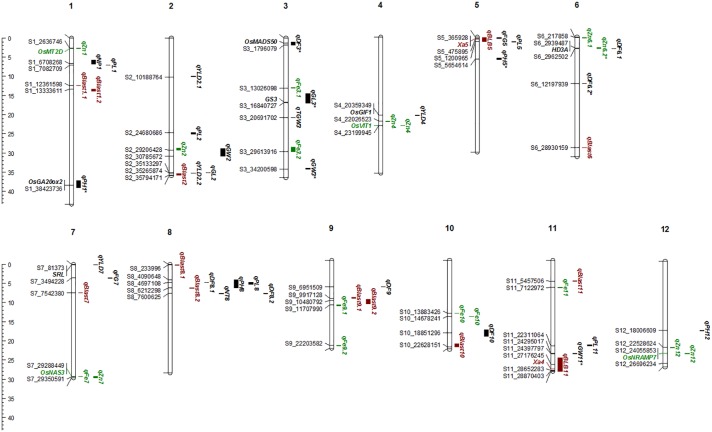
The Physical map of QTLs and genes for agronomic, biofortification, and disease resistance traits identified in the MAGIC Plus population. DF, days to 50% flowering; PH, plant height (cm); NT, number of tillers; NP, number of panicles; PL, panicle length (cm); GL, grain length (mm); GW, grain width (mm); FG, number of filled grains; TGW, thousand grain weight (g); YLD, grain yield (Kg/ha); Fe, iron (ppm); Zn, zinc (ppm); All the QTLs for agronomic traits highlighted in black, for biofortification in green and for diseases resistance in brown color. The scale on the left side indicates the distance in megabases (mb).

A total of 30 QTLs were identified for the ten agronomic traits (**Figure 5** and **Table 4**). Manhattan plots showing significant SNP peaks for different traits along with their respective QQ plots are presented (**Supplementary Figures S1**–**S10**). QTLs that were uncovered in at least two environments include *qDF*_3.1_, *qDF*_6.2_, and *qDF*_9.1_, *qPH*_1.1_ and *qPH*_5.1_, *qGL*_3.1_, *qGW*_3.1_, and *qGW*_11.1_. Moderate-effect (>10% PVE) and large-effect QTLs identified in specific environments were *qDF*_10.1_, *qPH*_8.1_, *qPH*_12.1_, *qNT*_8.1_, *qPL*_5.1_, *qPL*_8.1_, *qYLD*_2.1_, *qYLD*_6.1_, *qYLD*_4.1_, and *qYLD*_7.1_.

**Table 4:** QTLs for agronomic, biofortification and disease resistance traits in the MAGIC Plus population.

Trait	QTL	Peak SNP	Chr	Position (kb)	-log (*p*-Value)	PVE (%)	Environment
DF	*qDF*_3.1_	S3_1796079	3	1002–1796	4.7	14.9, 12.4	E1, E2
	*qDF*_6.1_	S6_2962502	6	2963	3.3	9.3	E2
	*qDF*_6.2_	S6_12197939	6	12198	3.8	11.8,10.0	E1, E2
	*qDF*_8.1_	S8_4697108	8	4697	3.3	10.1	E2
	*qDF*_8.2_	S8_7600625	8	7601	3.3	10.4	E2
	*qDF*_9.1_	S9_6951509	9	6952	3.3	9.2, 8.5	E1, E2
	*qDF*_10.1_	S10_18851296	10	18116–19895	4.3	13	E1
PH (cm)	*qPH*_1.1_	S1_38423736	1	37162–38997	8.2	29.6, 19.0, 17.0, 20.4	E1, E2, E3, E4
	*qPH*_5.1_	S5_5654614	5	5448–5848	4.1	12.5, 10.3	E1, E2
	*qPH*_8.1_	S8_4090648	8	4091–6230	4.6	14.1	E2
	*qPH*_12.1_	S12_18006609	12	18017	4.7	14.6	E2
NT	*qNT*_8.1_	S8_7600625	8	7601	4.1	12.5	E1
NP	*qNP*_1.1_	S1_6708268	1	5657–6788	3.7	11	E2
PL (cm)	*qPL*_1.1_	S1_7082709	1	7083	3.7	10.6	E2
	*qPL*_2.1_	S2_24680686	2	24.681–25077	3.8	11.1	E1
	*qPL*_5.1_	S5_1200965	5	1201	4.1	12.7	E1
	*qPL*_8.1_	S8_4697108	8	4697–5217	5.9	18.8	E2
	*qPL*_11.1_	S11_22311064	11	22069–22311	3.8	11.6	E2
FG	*qFG*_5.1_	S5_365928	5	163–411	3.8	11.4	E1
	*qFG*_7.1_	S7_3494228	7	3494–3609	3.3	9.5	E2
GL (mm)	*qGL*_2.1_	S2_35133297	2	35133	3.4	9.9	E2
	*qGL*_3.1_	S3_16840727	3	14528–17044	9.3	32.2, 33.3	E1, E2
GW (mm)	*qGW*_2.1_	S2_30785672	2	28899–30785	3.5	10.8	E2
	*qGW*_3.1_	S3_34200598	3	33986–34201	3.8	11.1, 8.43	E1, E2
	*qGW*_11.1_	S11_24295017	11	24295–24398	3.4	9.9, 12.3	E1, E2
TGW (g)	*qTGW*_3.1_	S3_20691702	3	20692	3.3	9.6	E2
YLD (Kg/ha)	*qYLD*_2.1_	S2_10083985	2	9976–10084	3.7	12.2	E3
	*qYLD*_2.2_	S2_35265874	2	35266	4.0	11.8	E2
	*qYLD*_4.1_	S4_20359349	4	20359–20468	3.5	10.7	E4
	*qYLD*_7.1_	S7_81373	7	45–134	3.6	10.9	E4

Fe (ppm)	*qFe*_3.1_	S3_13026098	3	12926–13026	3.3	9.9	E4
	*qFe*_3.2_	S3_29613916	3	28530–29663	3.3	9.5	E3
	*qFe*_7.1_	S7_29288449	7	29281–29288	3.4	10	E1
	*qFe*_9.1_	S9_11707990	9	11707–11930	3.8	10.9	E1
	*qFe*_9.2_	S9_22203582	9	22186–22415	4.5	13.7	E1
	*qFe*_10.1_	S10_13883426	10	13883–14678	3.3	9.5,11.6	E2, E4
	*qFe*_11.1_	S11_7122972	11	7123	3.3	9.3	E3
Zn (ppm)	*qZn*_1.1_	S1_2636746	1	2637	3.3	9.4	E4
	*qZn*_2.1_	S2_29206428	2	28727–29206	3.8	11.6	E2
	*qZn*_4.1_	S4_22026522	4	22027–23200	3.2	9.2, 9.5	E2, E4
	*qZn*_6.1_	S6_217858	6	218	3.4	10.4	E3
	*qZn*_6.2_	S6_2939487	6	2785–2963	3.8	10.9, 10.4	E2, E3
	*qZn*_7.1_	S7_29350591	7	29288–29624	4.1	10.1, 13.7, 11.2, 13.75	E1, E2, E3, E4
	*qZn*_12.1_	S12_22528624	12	22529–24056	3.3	10.1, 9.8	E1, E2
PXO61	*qBLB*_11.1_	S11_27496928	11	20064–28967	18.4	24.6	GH
PXO99	*qBLB*_11.1_	S11_27183806	11	27176–28920	5.2	14.3	GH
PXO341	*q BLB*_11.1_	S11_28870403	11	20064–28967	17.4	62	GH
PXO86	*qBLB*_5.1_	S5_475895	5	79–1224	8.1	24.6	GH
Blast	*qBlast*_1.1_	S1_12361598	1	12350–12362	3.6	10	GH
	*qBlast*_1.2_	S1_13333611	1	13334–13798	3.3	8.8	GH
	*qBlast*_2.1_	S2_35794171	2	35357–35794	3.9	11.9	GH
	*qBlast*_6.1_	S6_28930159	6	28927–28930	3.4	9.2	GH
	*qBlast*_7.1_	S7_7542380	7	7542	3.0	9.2	GH
	*qBlast*_8.1_	S8_233996	8	234	3.8	10.4	GH
	*qBlast*_8.2_	S8_6212298	8	6212	3.9	10.6	GH
	*qBlast*_9.1_	S9_9917128	9	9675–9917	3.6	8.3	GH
	*qBlast*_9.2_	S9_10480792	9	10161–11269	3.1	9.8	GH
	*qBlast*_10.1_	S10_22628151	10	21804–22628	4.0	13.2	GH
	*qBlast*_11.1_	S11_5457506	11	5458	3.3	9.6	GH


*DF, days to 50% flowering; PH, plant height (cm); NT, number of tillers; NP, number of panicles; PL, panicle length (cm); GL, grain length (mm); GW, grain width (mm); FG, number of filled grains; TGW, thousand grain weight (g); YLD, grain yield (Kg/ha); Fe, iron (ppm); Zn, zinc (ppm); QTL, quantitative trait loci; kb, kilobase; SNP, single nucleotide polymorphism; PVE, phenotypic variance explained (%); Chr, chromosome; GH, green house; E, denotes environments.*

A number of genes identified either within or near QTLs in this study have functions relevant to the associated traits (**Supplementary Table S1**). The QTLs *qDF*_3.1_ and *qDF*_6.1_ co-located with *OsMADS50* and *HD3A*, respectively, which are known major genes for days to flowering. *OsMADS50* is an important activator of flowering in rice while *HD3A* is related to photoperiod sensitivity (Yano et al., 2001). The QTL for plant height (*qPH*_1.1_) was co-located with *OsGA20ox2/sd1* while *qPH*_8.1_ was co-located with *OsBAK1*. The QTL *qGL*_3.1_ corresponds to *GS3*, a major gene controlling grain length. Meanwhile, QTLs *qYLD*_4.1_ and *qYLD*_7.1_ co-located with *OsGIF1* and *SRL1*, respectively.

#### Biofortification Traits

**FIGURE 6:**
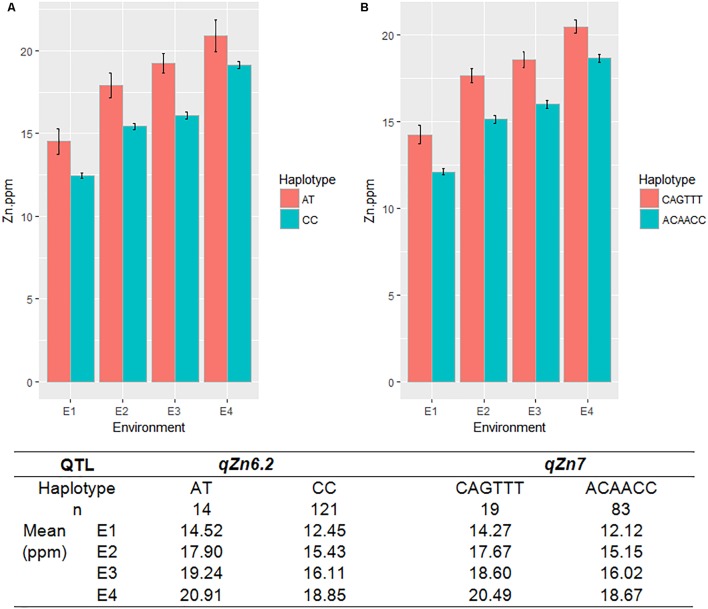
The haplotypes in the major effect QTLs for grain Zn, *qZn*_6.2_ **(A)** and *qZn*_7_ **(B)**, and their effect on Zn phenotype in different environments. Red and green color indicates different haplotypes within the QTLs, E-denotes different environments, *n*-indicates the number of MAGIC lines with different haplotypes.

A total of seven QTLs each were identified for Fe and Zn (**Table 4**, **Figure 5**, and **Supplementary Figures S11**, **S12**). Moderate-effect QTLs (>10% PVE) identified in specific environments were *qFe*_7.1_, *qFe*_9.1_, and *qFe*_9.2_. Two QTLs for Zn on chromosomes 6 and 7 were consistent in at least two environments which were represented by eight marker-Zn associations (**Table 5**). In all cases, high Zn was associated with the minor alleles. Substantial LD (*r*^2^ > 0.35) was also observed among associated markers within both QTLs. Haplotypes for both QTLs were further examined (**Figure 6**). Only two haplotypes, AT and CC, of *qZn*_6.2_ were observed in the population indicating that there is tight linkage between the two loci. The AT haplotype is associated with high Zn. Meanwhile, the two most common haplotypes in *qZn*_7.1_ were ACAACC and CAGTTT. The latter was associated with high Zn. The stability of the markers in these loci has clearly shown its potential for use in biofortification breeding programs although further validation is still necessary.

**Table 5:** SNPs consistently associated with grain Zn in at least two environments.

Marker	Allele^a^	Chromosome	Position	Env
S6_2939487	A	6	2939487	E2, E3
S6_2962502	T	6	2962502	E2, E3
S7_29281096	C	7	29281096	E2, E3
S7_29288449	A	7	29288449	E2, E3, E4
S7_29340541	G	7	29340541	E1, E2
S7_29350591	T	7	29350591	E1, E2, E3, E4
S7_29489905	T	7	29489905	E2, E4
S7_29623625	T	7	29623625	E1, E2


*^*a*^minor allele associated with high Zn; A, adenine; T, thymine; C, cytosine; G, guanine.*

Several known genes for metal transport and homeostasis were located either within or near two Fe QTLs and five Zn QTLs. The gene *MTP6*, a cation transmembrane transporter, is located within the LD block of QTL *qFe*_3.1_. *OsMT2d*, a metallothionein gene, is located near *qZn*_1.1_, *OsVIT1* gene is located near *qZn*_4.1_, and *OsNRAMP7* is near *qZn*_12.1_. Finally, *OsNAS3* is located within *qFe*_7.1_ and *qZn*_7.1_.

#### Disease Resistance Traits

A total of two major-effect (>25% PVE) QTLs were identified for resistance against four races of BLB while a total of ten QTLs were identified for blast disease resistance (**Table 4**, **Figure 5**, and **Supplementary Figures S13**, **S14**). The QTLs identified for resistance against *PXO61, PXO99*, and *PXO341* co-located on chromosome 11 while the QTL for resistance against *PXO86* was identified on chromosome 5.

### Bayesian Network Analysis

**FIGURE 7:**
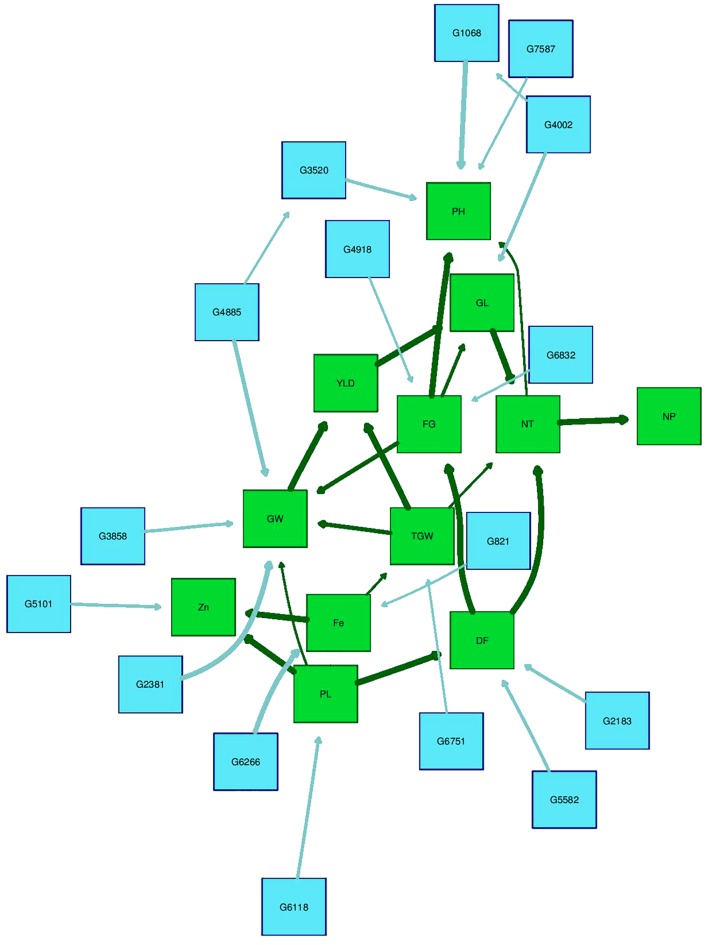
Bayesian network of agronomic and biofortification traits observed in the MAGIC Plus population using genotypic data and BLUPs values from all environments. DF, days to 50% flowering; PH, plant height (cm); NT, number of tillers; NP, number of panicles; PL, panicle length (cm); GL, grain length (mm); GW, grain width (mm); FG, number of filled grains; TGW, thousand grain weight (g); YLD, grain yield (Kg/ha); Fe, iron (ppm); Zn, zinc (ppm); All labels with ‘G’ are the SNPs, The arrows indicate the direction of influence and thickness of the arrow indicates the degree of influence among different traits and with the SNPs.

The Bayesian network analysis was carried out using the BLUPs of different traits from all the environments. The averaged BN at a significance level of α = 0.01 shows the strength and direction of the relationship among traits and markers in all environments (**Figure 7**). There were 28 nodes and 36 arcs among 12 traits and 16 markers in the overall BN. The SNP markers corresponding to the encoded labels in the figures are shown in **Table 6**. There were 18 trait-trait relationships, 16 marker-trait relationships, and two marker-marker relationships. BN showed very complex relationships among different traits, some of them with significant direct influence were between DF→FG, DF→NT, PL→DF, PL→Zn, TGW→GW, GW→YLD, Fe→Zn, FG→PH, YLD→FG, GL→NT and NT→NP. Further, only four of the marker-trait relationships identified in BN were consistent with marker-trait associations uncovered by GWAS. Markers G2183 and G1068 correspond to *qPH*_1.1_ and *qDF*_3.1_, respectively, while markers G6266 and G5101 correspond to *qFe*_10.1_.

**Table 6:** SNP markers significantly associated with different traits in the Bayesian analysis.

Label	SNP Marker	Label	SNP Marker
G821	S1_25071488	G4918	S7_22935899
G1068	S1_38423736	G5101	S7_29288449
G2183	S3_1796079	G5582	S8_26508206
G2381	S3_16975678	G6118	S10_4326881
G3520	S4_31442950	G6266	S10_13883426
G3858	S5_15133087	G6751	S11_15486699
G4002	S5_28279181	G6832	S11_18743527
G4885	S7_22450212	G7587	S12_25983316


*SNP, single nucleotide polymorphism.*

### *In silico* Analysis for Candidate Genes Underlying Novel Fe, Zn, and Blast Resistance QTLs

To identify additional novel candidate genes, the comprehensive set of genes underlying the new QTLs for Fe and Zn biofortification as well as new blast resistance QTLs were identified in the context of the gold-standard *japonica* reference genome Nipponbare (IRGSP 1.0) and two newly published high-quality genomes from the *indica* variety group Minghui 63 and IR8 (Zhang et al., 2016). Of the 17 QTLs mapped to Nipponbare, 15 of them were common to the IR8 genome and 11 QTLs were common to Minghui 63 (**Supplementary Table S2**). Comparing the genes annotated within the equivalent QTLs between the three reference genomes (**Supplementary Table S3**), it is interesting to note the following: (1) the number of genes in the equivalent QTL regions differs (1,100 in Nipponbare, 773 in IR8, 708 in Minghui 63), (2) the genes within the equivalent QTLs are not the same (469 of 773 IR8 genes and 397 of 708 Minghui 63 genes are common to Nipponbare), and (3) there are unique (non-Nipponbare) genes in the *indica* genomes within these QTLs. The list and annotation of IR8 and Minghui 63 genes (common with Nipponbare, unique from Nipponbare) as well as the un-annotated genes are provided in **Supplementary Table S3**.

SNP analysis of IR69428-6-1-1-3-3 and IR64 in the Zn homeostasis genes showed that there are some interesting changes in the promoter and coding regions of genes over expressed. The list of polymorphic SNPs is presented in the **Supplementary Table S4**. There were 12 polymorphic SNPs each in *OsNAS1* and *OsNAS2*, 15 in *OsNAS3*, 16 SNPs in *OsNRAPM7* and none were polymorphic for *OsVIT1*.

### Expression Analysis of Fe and Zn Homeostasis Genes

**FIGURE 8:**
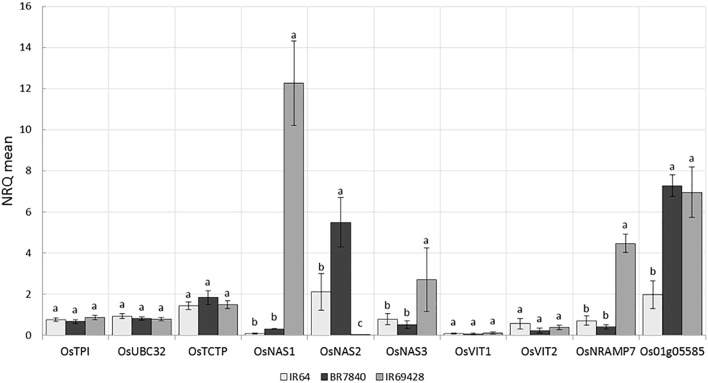
Expression levels of reference and target genes in grains at 11 DAF (a) and 15 DAF (b) (5 biological replicates constituting of pooled seeds from five panicles or of a pool of 5 flag leaves were sampled and frozen in liquid nitrogen. Tissues were ground and total RNA extracted and used for qPCR analysis). The X-axis shows the genes and the Y-axis shows the level of expression in three rice genotypes.

IR64 (low Zn) and IR69428 (high Zn) are presented in the current study. Gene expression in grain of different varieties at different grain-filling time points, through RT-PCR for key genes found in the literature and through the above QTL and whole-transcriptome analyses. The expression levels of Zn homeostasis genes in rice were examined using a set of 24 validated genes for metal uptake, transport, and translocation. The expression studies confirm that *OsNAS* genes are involved in elevated levels of Zn. We found at 11 days after flowering that the grains from IR69428 (Zn-dense donor) show a 12-fold difference from the levels in IR64 for *OsNAS1* and a 2-fold difference for *OsNAS3* (**Figure 8**). Another genotype, BR7840 (Zn-dense genotype), shows up-regulation of *OsNAS2*. We also saw that the expression levels of *OsNRAMP7* and *metallothionein*/*OsMT2d* genes were higher at 11 DAF in grain of IR6948 than in IR64 (**Figure 8**). The expression levels of most genes fall at 15 DAF and we did not see significant differences between genotypes (**Figure 8**).

## Discussion

Breeding rice varieties with enhanced nutrition, especially for Fe and Zn along with a suite of desirable agronomic and diseases resistance traits is a major priority of rice research (Bouis, 2003; Swamy et al., 2016). In our study we characterized MAGIC Plus lines for agronomic, biofortification and diseases resistance traits; mapped QTLs and identified gene pyramided lines with different combination of desirable traits.

The phenotypic analysis of MAGIC Plus lines showed wide variations for all the traits and most of them exhibited normal distributions indicating their polygenic inheritance. The heritability values were moderate to high (H^2^ > 45%) for most of the traits except for NP and Fe. A high H^2^ (>50%) and significant genotype and environment interactions (GxE) for agronomic traits and mineral elements, such as Fe, Zn, Cu, Mo, and Mg, observed across the populations and locations in most the studies on grain micronutrients (Stangoulis et al., 2007; Garcia-Oliveira et al., 2009; Norton et al., 2010, 2014; Bekele et al., 2013; Du et al., 2013; Sala et al., 2013; Gande et al., 2014; Huang et al., 2015; Xu et al., 2015).

There were many positive correlations among different agronomic traits but YLD was negatively correlated with Fe and Zn; while Fe and Zn were always strongly positively correlated irrespective of the environments and populations. Several earlier studies have reported the positive relationship between Fe and Zn as well as the negative relationship between YLD and Zn in rice (Norton et al., 2010, 2014; Bekele et al., 2013; Sala et al., 2013; Gande et al., 2014). However, there are some reports showing positive or no significant correlations between yield and Zn (Rai et al., 2012; Gangashetty et al., 2013; Sathisha, 2013). This indicates that it is possible to select high-Zn rice lines without any yield penalty. A positive correlation between Fe and Zn could allow simultaneous improvement of both minerals by breeding. However, the negative genetic linkages between YLD and Zn must be broken for successful Zn biofortification of rice. Hence it is necessary to identify high-Zn parental lines with acceptable yield potential, and to develop high Zn pre breeding and elite breeding lines by designing appropriate breeding strategies for the successful development and release of high-Zn rice varieties (Swamy et al., 2016).

### GWAS Identified Common and Environment Specific QTLs in MAGIC Plus Lines

As the population was tested in different locations and seasons, the stability of QTLs across environments was also explored. Although majority of the significant QTLs were detected in only one environment, several were detected in more than one environment and also showed stability in terms of genetic effects. Among five traits which were measured in all the four environments only for PH and Zn, QTLs *PH*_1.1_ and *Zn*_7.1_ were consistently identified in all the four environments. Ten QTLs such as *qDF*_3.1_, *qDF*_6.2_, *qDF*_9.1_, *qPH*_5.1_, *qGL*_3.1_, *qGW*_3.1_, *qGW*_11.1_, *qFe*_10.1_, *qZn*_6.2_ and *qZn*_12.2_ were detected in two environments, while two major loci *qBLB*_11.1_ and *qBLB*_5.1_ were identified for BLB resistance and several loci were identified for blast resistance. Eventhough mapping populations used in this study was smaller (144 lines) compared with the sample size used in other GWAS studies using MAGIC populations (Bandillo et al., 2013; Meng et al., 2016), we could successfully detect previously reported major genes and QTLs for agronomic, biofortification, and disease resistance traits (Huang et al., 2010; Han and Huang, 2013; Yang et al., 2014; Wu et al., 2015). This clearly indicates the utility of MAGIC lines for mapping QTLs and genes more precisely and accurately for both simple and complex traits, and also to develop gene/QTL pyramided lines with multiple desirable traits.

A number of correlated traits were associated with the same QTLs in their respective environments. In E1, the positively correlated Fe and Zn shared a common QTL, *qFe*_7.1_ and *qZn*_7.1_. In E2, positive correlations were uncovered between DF and Zn, and PH and PL. The QTL *qDF*_6.1_ overlapped with *qZn*_6.2_. QTLs *qPH*_8.1_ and *qPL*_8.1_ have three common SNP markers. However, some traits also shared the same QTL but were not correlated such as DF and PH, and GW and Zn, in E2. QTLs *qDF*_8.1_, *qPH*_8.1_, *qPL*_8.1_ and *qBlast*_8.2_ had a common SNP marker while *qGW*_2.1_ and *qZn*_2.1_ overlapped in E2; *qGL*_2.1_, *qYLD*_2.2_ and *qBlast*_2.1_ in E2, *qPL*_5.1_ and *qBLB*_5.1_; *qFe*_9.1_ and *qBlast*_9.2_ in E1 were co-located. The QTLs co-location with positive effect on multiple traits will be beneficial for their simultaneous improvement. However, QTLs with negative linkages and having opposite effects on the traits have to be carefully eliminated through prebreeding before using them in Marker Assisted Breeding.

It is interesting to note that 30 of the 57 QTLs identified in this study either included the known candidate genes or were near already identified QTL regions (**Supplementary Table S1**). The associated SNP markers of these major QTLs were found to co-locate with known major genes such as *OsMADS50* for DF, *osGA20ox2* for PH, *GS3* for GL and*OsNRAMP7*, *OsVIT*, *OsNAS3* for Zn, (Spielmeyer et al., 2002; Lee et al., 2004; Fan et al., 2006; Johnson et al., 2011; Zhang et al., 2012). The significant SNP markers associated with BLB and blast resistance were also either co-localized with or flanked by known resistance genes such as *Xa4* and *xa5* for BLB resistance, and *Pi28(t)*, *Pi30(t)*, and *Pi32(t)* for blast resistance (Sallaud et al., 2003; Niño-Liu et al., 2006; Liu et al., 2011). In addition, GWAS also detected novel QTLs for blast disease resistance that will be able to contribute further to disease resistance breeding programs. Since most of the QTLs are season and location specific, and also found to have genetic back ground effect it will be necessary to pool multiple loci so that marker- assisted QTL pyramiding, marker-aided recurrent selection, or genomic selection to develop rice varieties with improved micronutrient content.

### Bayesian Network Analysis Results Support Phenotypic Correlations and GWAS Result

Overall, BN analysis revealed valuable insights into interactions among phenotypes and SNP markers and to establish relationships among them. One-fourth of significant marker-trait relationships detected in BN analysis were consistent with the GWAS results including marker-trait associations for PH, DF, Zn, and Fe. Among trait relations most of the yield and yield components were highly interconnected but GW and TGW had very significant direct influence on YLD. Some significant direct influences among different trait combinations included NT → NP. It is also interesting to note that none of the agronomic traits except PL had any direct effect on grain Fe and Zn. While, Fe and Zn concentrations had very good association providing opportunities for simultaneous improvement of both the minerals. But there was no direct effect of YLD on Zn in the BNs indicating that yield and Zn can be combined to develop high Zn rice varieties. However, the effect of yield components on grain Zn through inter connections of PL needs further genetic dissection. YLD can exert a significant dilution effect on Zn, which may result in negative correlations between these traits such as in this study. Correcting for yield to remove its effect when evaluating for Zn may be necessary if negative association exists between them (McDonald et al., 2008). However, YLD can have a positive correlation or lack correlation with Zn, requiring no corrective measures (Nagesh et al., 2012; Sala and Geetha, 2015).

### Expression Analysis Showed Upregulation of Candidate Genes for Biofortification Traits

Genome-wide association studies identified SNP markers near known genes with functions related to Fe and Zn homeostasis. These genes were *OsMT2d, OsMTP6, OsVIT1, OsNAS3*, and *OsNRAMP7.* Quantitative expression analysis revealed up regulation of *OsNAS1* and *OsNAS3* in the grains of IR69428 and upregulation of *OsNAS2* in the BR7840 at 11DAF in comparison with IR64 (**Figure 8**). The expression study confirms that *OsNAS* genes are involved in elevated levels of Zn. At the same sampling point, expression levels of metallothionein (*Os01g05585*) in both donor genotypes were significantly different from the check variety. Our results confirm findings from previous studies that the over expression of *OsNAS3* and *OsNAS2* resulted in elevated levels of Fe and Zn in the seeds specifically in the endosperm of rice (Lee et al., 2009, 2011; Johnson et al., 2011). We further need to identify SNPs in these candidate genes and validate them on mapping populations. We also observe distinct clustering of expression values of target genes analyzed from IR69428 grains at 11 DAF from the rest of the samples. Moreover, over-expression of *AtNAS1* and *Pvferritin* in rice elevated Zn concentration in the grains (Wirth et al., 2009). *OsVIT1* is a vacuolar membrane transporters mainly expressed in the leaf and seeds whose functional disruption resulted in an elevated accumulation of grain Zn and reduction of these micronutrients in the leaf tissues (Zhang et al., 2012). Over-expression of *OsNRAMP7, OsNAS1*, and *OsFRO1* in the flag leaves showed significant correlations with Zn concentration in the seeds (Sperotto et al., 2010). Further, expression of *OsVIT1* and *OsNRAMP7* genes in the flag leaf was highly correlated with high grain Zn content (Banerjee and Chandel, 2011). Moreover, *OsFRO1* was up-regulated in roots and flag leaves of high-Zn rice lines (Chadha-Mohanty et al., 2015). *OsNAS* family genes are involved in biosynthesis, transport, and secretion of root exudates that increase metal uptake from the soil (Johnson et al., 2011; Nozoye et al., 2011) while *OsVIT1* is an important vacuole metal transporter involved in Zn transport across the tonoplast and also helps in Zn sequestration within the cell (Zhang et al., 2012). Nicotianamine synthesized by Nicotianamine synthase genes (*OsNAS*) as well as metallothioneins are involved in metal transport and homeostasis in plants (Douchkov et al., 2005; Yang et al., 2009). These genes should be further explored to develop gene based SNP markers for breeding high Zinc lines.

### *In silico* Analysis of Novel QTLs for Fe, Zn and Blast

The *in silico* sequence analysis within Fe and Zn QTLs lifted-over from Nipponbare to two *indica* reference genomes showed remarkable differences in the genes underlying QTL regions in different rice genomes, with 40–44% gene difference between Nipponbare and the two *indica* genomes. Of the genes unique to the *indica* reference genomes, 20–25% do not align to any partially non-redundant nucleotide sequences (NT database) from GenBank, EMBL, and DDBJ. This demonstrates the importance of using genome information in the reference genome that is most closely related to the parents used in the mapping population; the observed differences in the underlying genes of mapped QTLs across varietal groups imply that the “true” candidate gene(s) causative of QTL effects is(are) variety-specific, and also provides a hypothesis as to why some QTLs are varietal-background specific. Post-GWAS analyses such as candidate gene nomination from mapped QTLs should use information from the most closely related reference genome (or the genome sequence of the parents of the mapping population).

The *in silico* SNP analysis of the *OsNAS*, *OsNRAMP7*, *OsVIT1* genes between high Zn rice IR69428-1-1-1-6-3 and the check variety IR64 showed that there are several polymorphic functional SNPs both in the promoter and coding regions of these genes. However, these polymorphic SNPs have to be further validated to understand their role in grain Zn loading before using them in the rice biofortification breeding programs.

### MAGIC Lines With High Yield and High Grain Zn Were Identified

We identified nine MAGIC Plus lines with high grain Zn, acceptable yield potential along with BLB and blast resistance (**Table 2**). Two lines IR95095:3-B-11-19-8-GBS and IR95132:3-B-18-13-11-GBS had grain Zn > 18ppm and with a grain yield of >5.5 t (2015DS) and >3.0t/ha (2015WS). These lines can be used as donors in breeding programs or can be directly tested in multi-location trials to further evaluate their performance and release them as high Zn rice varieties. The MAGIC Plus lines derived from multiple parents and several cycles recombination’s lead to generation of elite breeding materials with gene pyramided lines for multiple traits. The results clearly showed that yield and grain Zn can be combined successfully and MAGIC Plus lines are a good resource for rice biofortification.

## Conclusion

The MAGIC Plus population in this study proved to be a useful mapping resource for agronomic, biofortification and disease resistance traits. Overall, 57 significant genomic regions were detected and 12 of them were consistent in more than two environments. Interestingly 30 of the 57 QTLs were co-located with major QTLs/genes such as *OsMADS50* for days to flowering, *osGA20ox2* for plant height, and *GS3* for grain length, *Xa4* and *xa5* for BLB resistance, *Pi5(t)*, *Pi28(t)* and *Pi30(t)* for Blast resistance. While, *OsMTP6*, *OsNAS3*, *OsMT2D*, *OsVIT1* and *OsNRAMP7* were co-located with QTLs for Fe and Zn. Bayesian analysis showed that agronomic and yield component traits had no significant direct effect on grain Fe and Zn, so yield and Zn can be combined to develop high Zn rice varieties. Gene expression analysis revealed up regulation of *OsNAS1, OsNAS3* and *OsNAS2* in high Zn donor lines. SNP analysis of promoter and coding regions of selected candidate genes in high Zn and control varieties showed interesting changes and needs further validation. The stability of markers surrounding *OsNAS3* gene has clearly shown its potential for use in biofortification breeding programs. On the other hand, the degree of association of significant markers for *Xa4* and *xa5* genes indicate that they are applicable for direct use in MAS. The high Zn lines with QTLs/genes pyramided and acceptable yield potential, which are a good resource for further evaluation to release as varieties as well as for use in breeding programs.

## Data Availability

The phenotypic data generated for this study has been provided as a **Supplementary Table S5**.

## Author Contributions

GD and HZ carried out the experiments, analyzed the data, and prepared the manuscript. MI-A, AA, and EA assisted in field experiments. RM carried out the bioinformatics analysis and edited the manuscript. PM carried out the gene expression analysis and edited the manuscript. CR and HL provided the MAGIC population, genotypic data, contributed to designing of the experiments, and reviewed the manuscript. JH, AL, MM, and MD reviewed the manuscript and provided critical comments. RR and BS conceptualized the study, supervised the experiments, and edited the manuscript. All the authors have read and approved the manuscript for publication.

## Conflict of Interest Statement

The authors declare that the research was conducted in the absence of any commercial or financial relationships that could be construed as a potential conflict of interest.

## Footnotes

1 http://rapdb.dna.affrc.go.jp/viewer/gbrowse/irgsp1

2 http://archive.gramene.org/qtl/

3 http://snp-seek.irri.org/

## Supplementary Material

FIGURE S1 Manhanttan plots and QQ-plots generated from genome-wide association study of days to flowering observed in the MAGIC Plus population in all environments. Highlighted genomic regions contain SNPs common to at least 2 environments (TIF).

FIGURE S2 Manhattan plots and QQ-plots generated from genome-wide association study of plant height observed in the MAGIC Plus population in all environments. Highlighted genomic regions contain SNPs common to at least 2 environments (TIF).

FIGURE S3 Manhattan plots and QQ-plots generated from genome-wide association study of number of tillers observed in the MAGIC Plus population in all environments (TIF).

FIGURE S4 Manhattan plots and QQ-plots generated from genome-wide association study of number of productive tillers observed in the MAGIC Plus population in all environments (TIF).

FIGURE S5 Manhattan plots and QQ-plots generated from genome-wide association study of panicle length observed in the MAGIC Plus population in all environments (TIF).

FIGURE S6 Manhattan plots and QQ-plots generated from genome-wide association study of grain length observed in the MAGIC Plus population in all environments. Highlighted genomic regions contain SNPs common to at least 2 environments (TIF).

FIGURE S7 Manhanttan plots and QQ-plots generated from genome-wide association study of grain width observed in the MAGIC Plus population in all environments. Highlighted genomic regions contain SNPs common to at least 2 environments (TIF).

FIGURE S8 Manhanttan plots and QQ-plots generated from genome-wide association study of filled grains observed in the MAGIC Plus population in all environments (TIF).

FIGURE S9 Manhanttan plots and QQ-plots generated from genome-wide association study of thousand grain weight observed in the MAGIC Plus population in all environments (TIF).

FIGURE S10 Manhattan plots and QQ-plots generated from genome-wide association study of yield observed in the MAGIC Plus population in all environments (TIF).

FIGURE S11 Manhattan plots and QQ-plots generated from genome-wide association study of grain iron concentration observed in the MAGIC Plus population in all environments (TIF).

FIGURE S12 Manhattan plots and QQ-plots generated from genome-wide association study of grain zinc concentration observed in the MAGIC Plus population in all environments. Highlighted genomic regions contain SNPs common to at least 2 environments (TIF).

FIGURE S13 Manhattan plots generated from MLM analysis of resistance to four races of bacterial leaf blight disease observed in the MAGIC Plus population (TIF).

FIGURE S14 Manhattan plots generated from MLM analysis of resistance to blast disease observed in the MAGIC Plus population (TIF).

TABLE S1 QTLs for agronomic, biofortification and disease resistance traits that co-locate with known genes/QTLs (CSV).

TABLE S2 QTL lift-over results (CSV).

TABLE S3 Counts of genes within lifted-over QTLs across three reference genomes (Nipponbare, IR8, Minghui 63) (XLSX).

TABLE S4 Polymorphic SNPs in the promoter and coding regions of the selected genes between high and low zinc rice lines.

TABLE S5 Phenoypic data used for the analyses.

## Abbreviations

Blast: blast
BLB: bacterial leaf blight
BN: Bayesian network
CV: co-efficient of variation
DF: days to 50% flowering
Fe: iron
FG: number of filled grains
GL: grain length
GW: grain width
GWAS: genome wide association analysis
H^2^: heritability
LD: linkage disequilibrium
MAGIC: multi parent advanced generation inter crosses
NAS: nicotianamine synthase
NP: number of panicles
NT: number of tillers
PH: plant height
PL: panicle length
QTL: quantitative trait loci
SNP: single nucleotide polymorphism
TGW: thousand grain weight
YLD: grain yield
Zn: zinc
