# Supplementary figures and images for: Genome-Wide Association Mapping in a Rice MAGIC Plus Population Detects QTLs and Genes Useful for Biofortification
Source: Front Plant Sci. 2018 Sep 20;9:1347. doi: 10.3389/fpls.2018.01347 (PMC6158342; doi:10.3389/fpls.2018.01347)

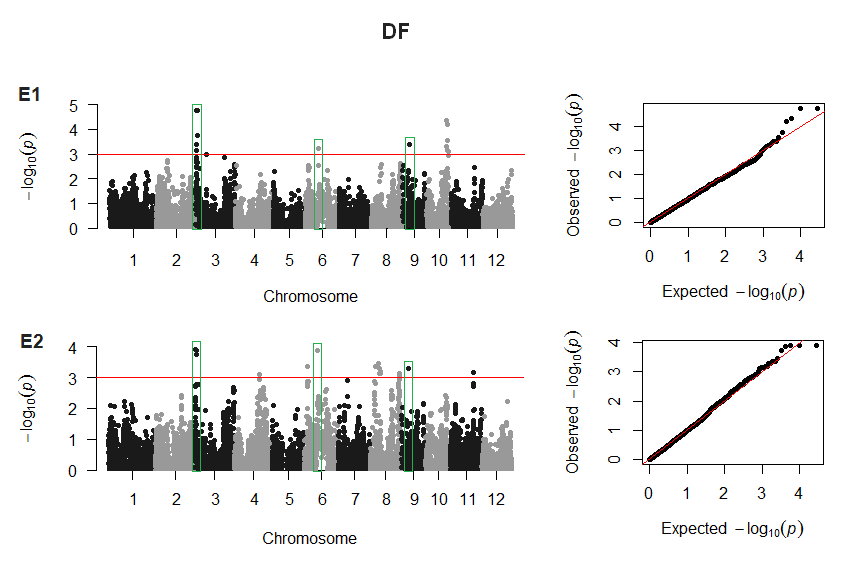

Supplement: FIGURE S1 — Manhanttan plots and QQ-plots generated from genome-wide association study of days to flowering observed in the MAGIC Plus population in all environments. Highlighted genomic regions contain SNPs common to at least 2 environments (TIF). [file Image_1.TIF]

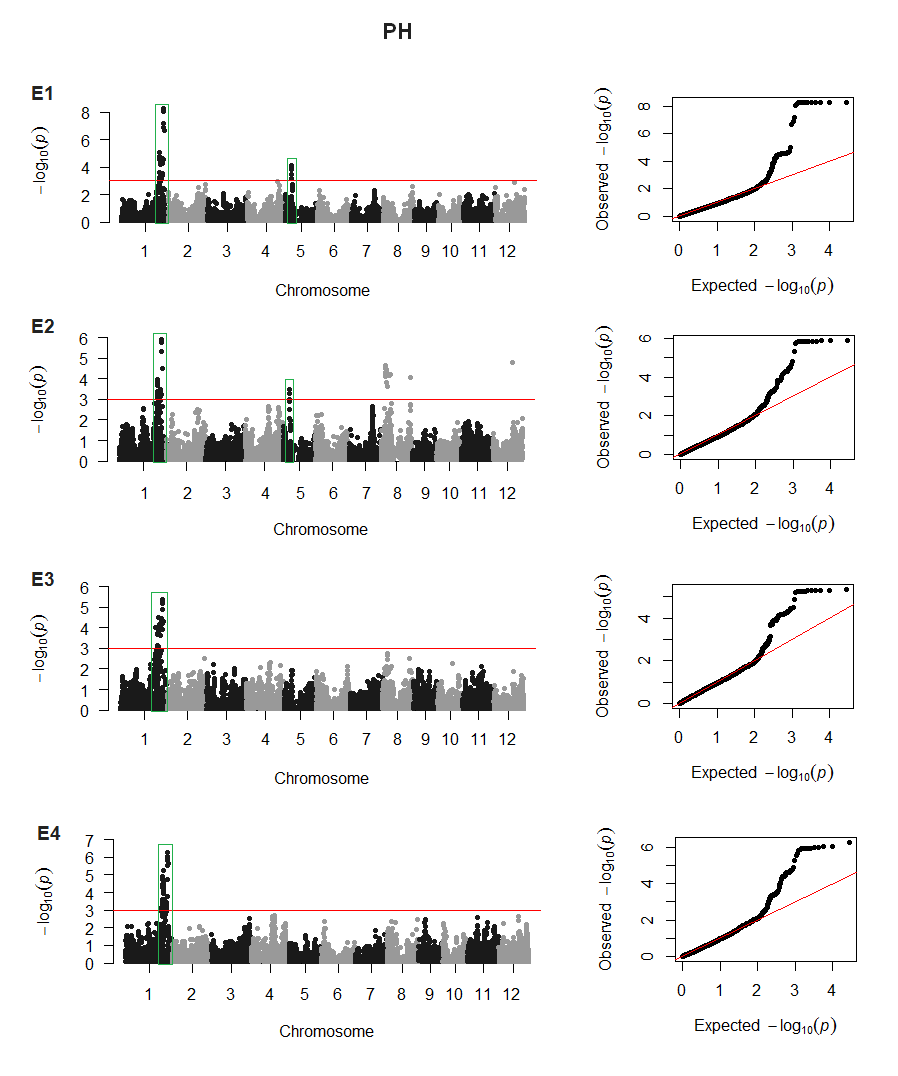

Supplement: FIGURE S2 — Manhattan plots and QQ-plots generated from genome-wide association study of plant height observed in the MAGIC Plus population in all environments. Highlighted genomic regions contain SNPs common to at least 2 environments (TIF). [file Image_2.TIF]

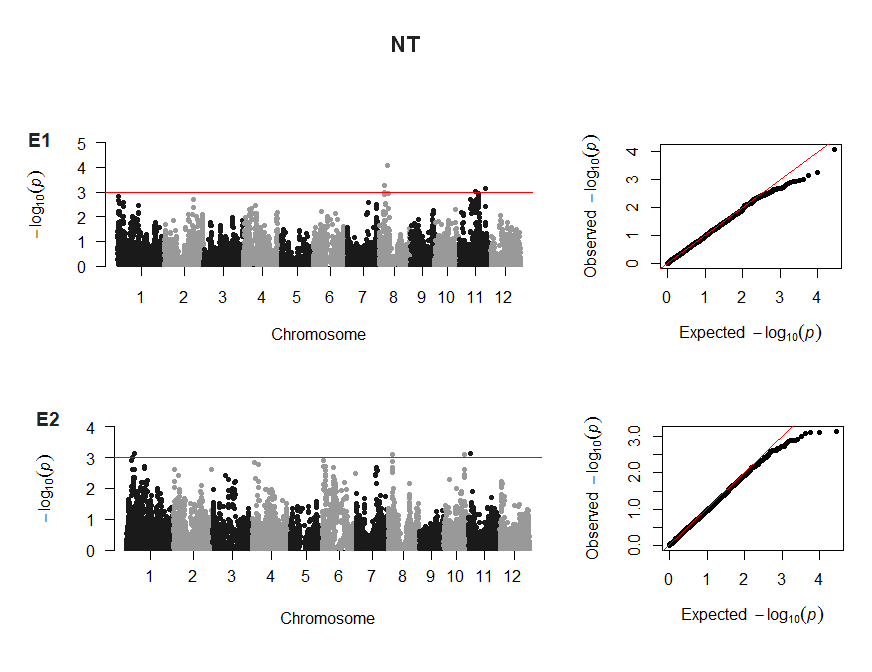

Supplement: FIGURE S3 — Manhattan plots and QQ-plots generated from genome-wide association study of number of tillers observed in the MAGIC Plus population in all environments (TIF). [file Image_3.TIF]

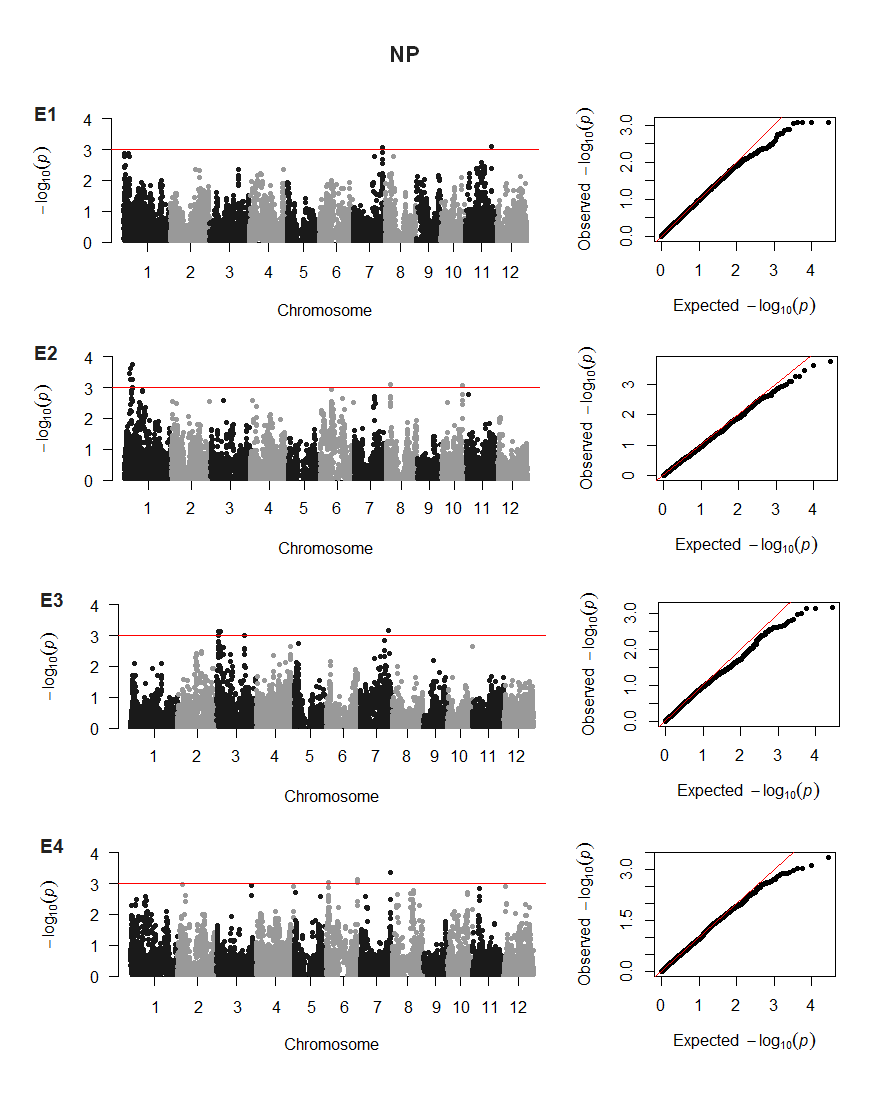

Supplement: FIGURE S4 — Manhattan plots and QQ-plots generated from genome-wide association study of number of productive tillers observed in the MAGIC Plus population in all environments (TIF). [file Image_4.TIF]

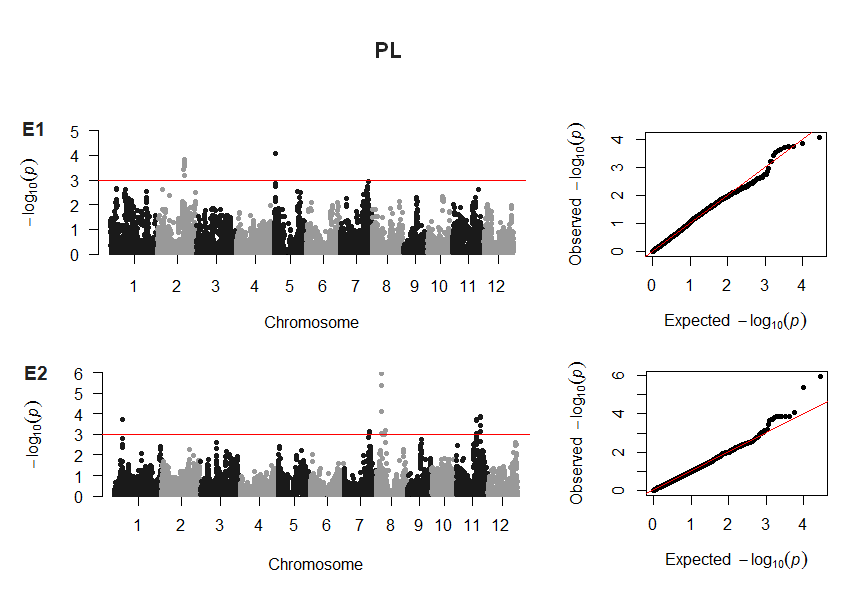

Supplement: FIGURE S5 — Manhattan plots and QQ-plots generated from genome-wide association study of panicle length observed in the MAGIC Plus population in all environments (TIF). [file Image_5.TIF]

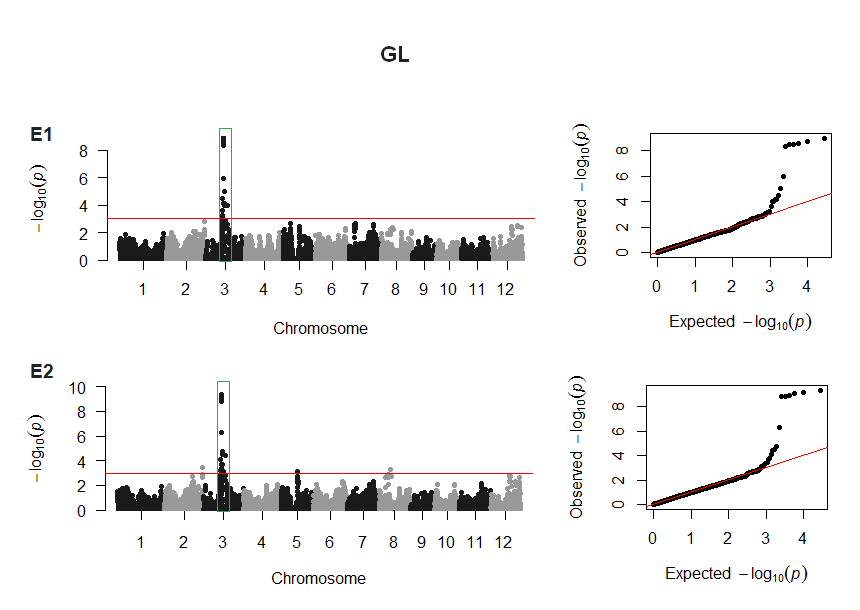

Supplement: FIGURE S6 — Manhattan plots and QQ-plots generated from genome-wide association study of grain length observed in the MAGIC Plus population in all environments. Highlighted genomic regions contain SNPs common to at least 2 environments (TIF). [file Image_6.TIF]

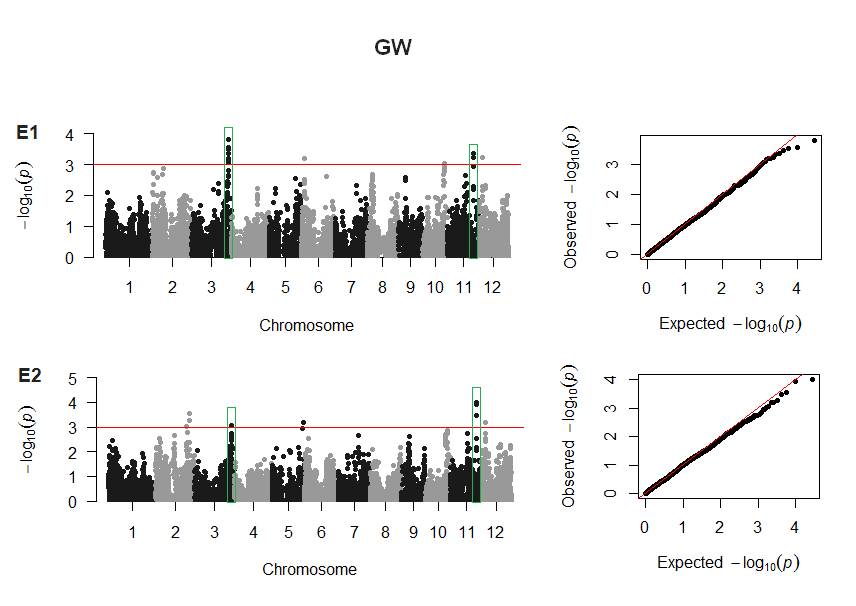

Supplement: FIGURE S7 — Manhanttan plots and QQ-plots generated from genome-wide association study of grain width observed in the MAGIC Plus population in all environments. Highlighted genomic regions contain SNPs common to at least 2 environments (TIF). [file Image_7.TIF]

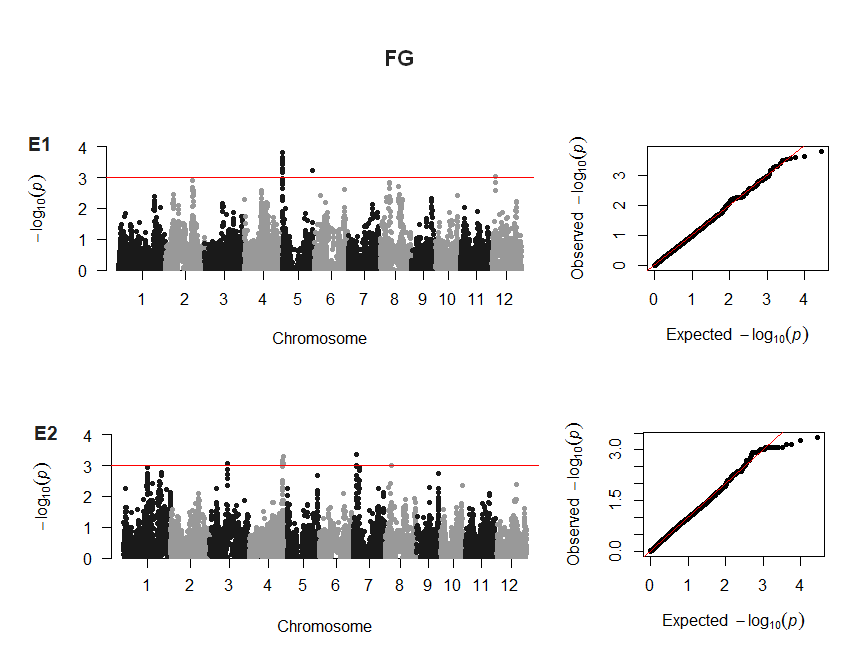

Supplement: FIGURE S8 — Manhanttan plots and QQ-plots generated from genome-wide association study of filled grains observed in the MAGIC Plus population in all environments (TIF). [file Image_8.TIF]

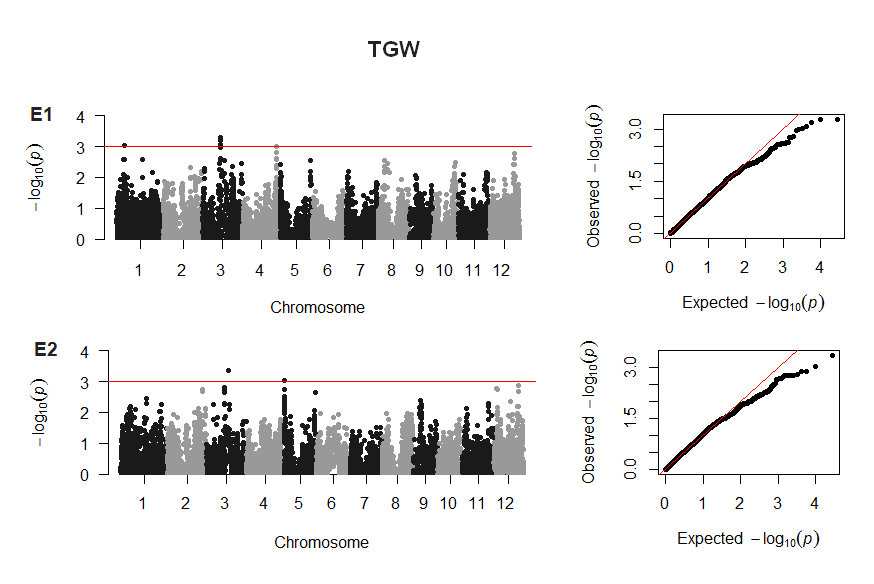

Supplement: FIGURE S9 — Manhanttan plots and QQ-plots generated from genome-wide association study of thousand grain weight observed in the MAGIC Plus population in all environments (TIF). [file Image_9.TIF]

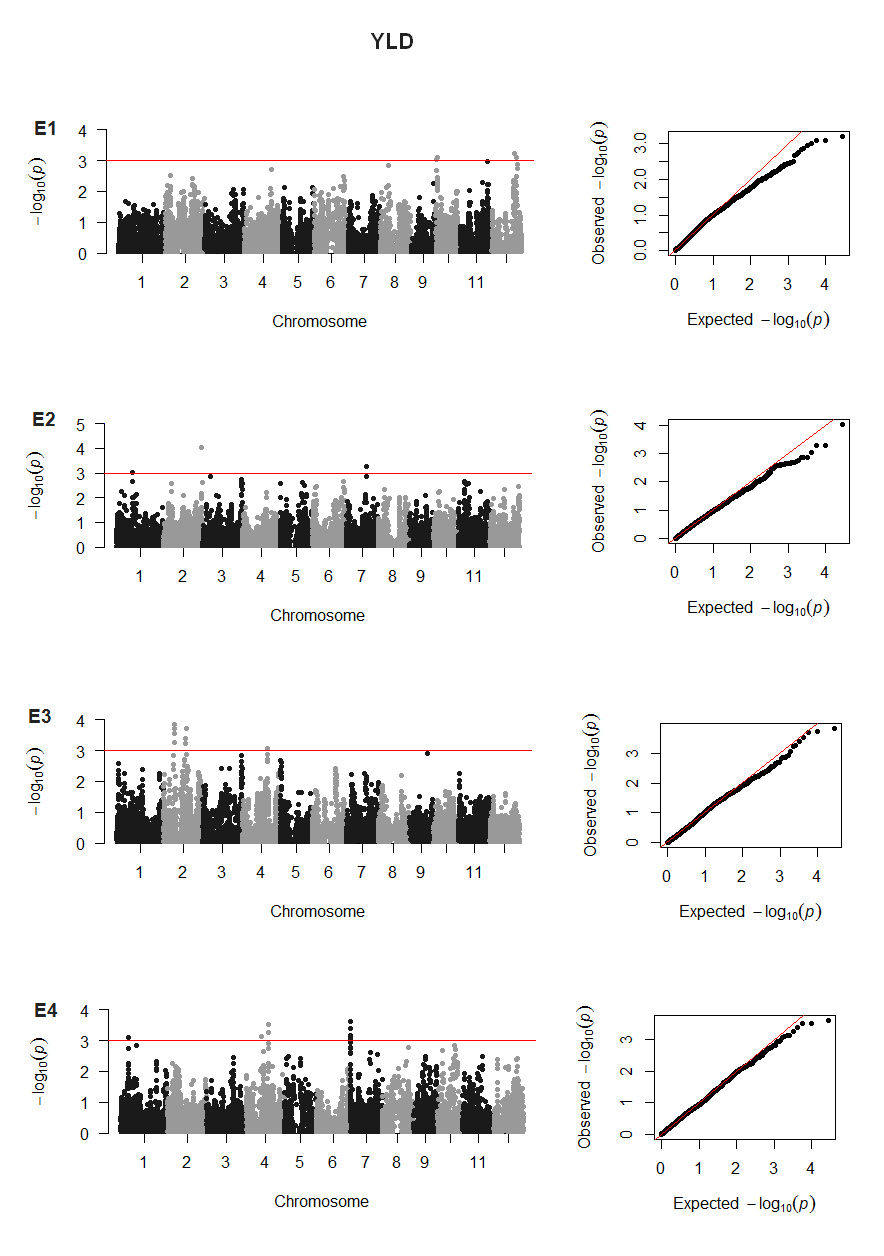

Supplement: FIGURE S10 — Manhattan plots and QQ-plots generated from genome-wide association study of yield observed in the MAGIC Plus population in all environments (TIF). [file Image_10.TIF]

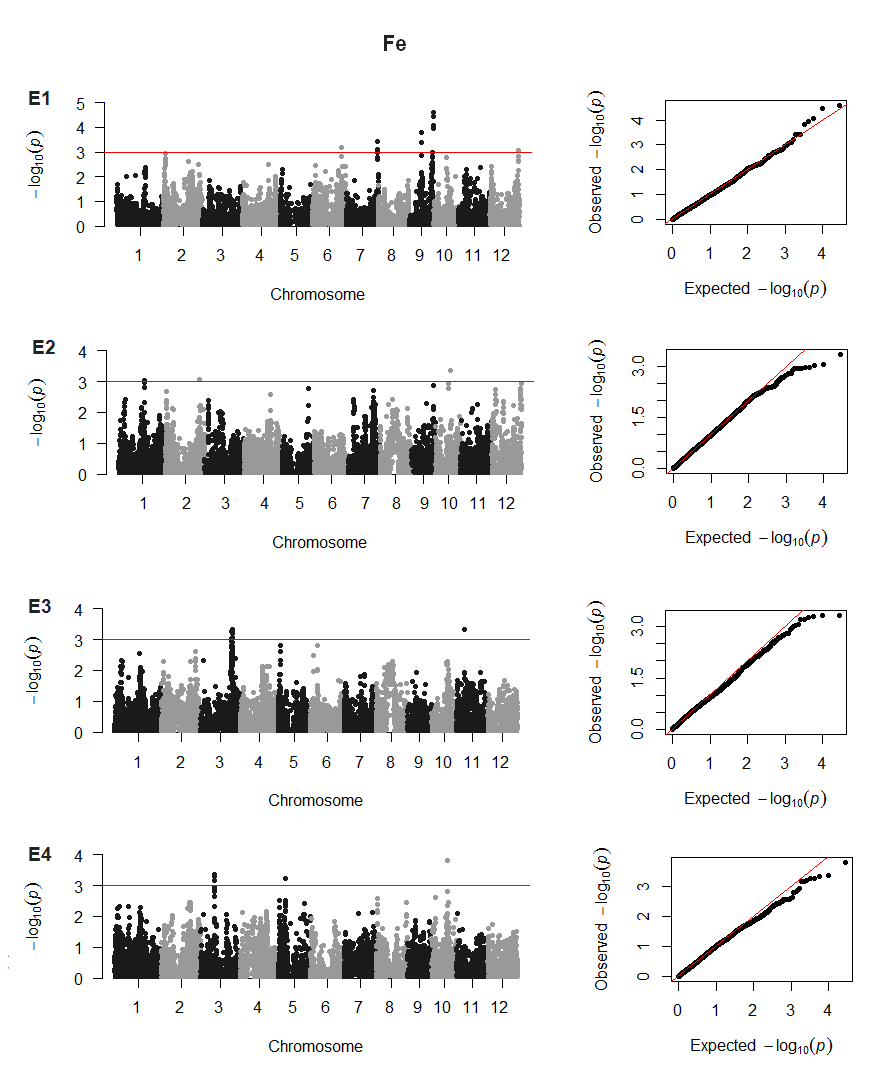

Supplement: FIGURE S11 — Manhattan plots and QQ-plots generated from genome-wide association study of grain iron concentration observed in the MAGIC Plus population in all environments (TIF). [file Image_11.TIF]

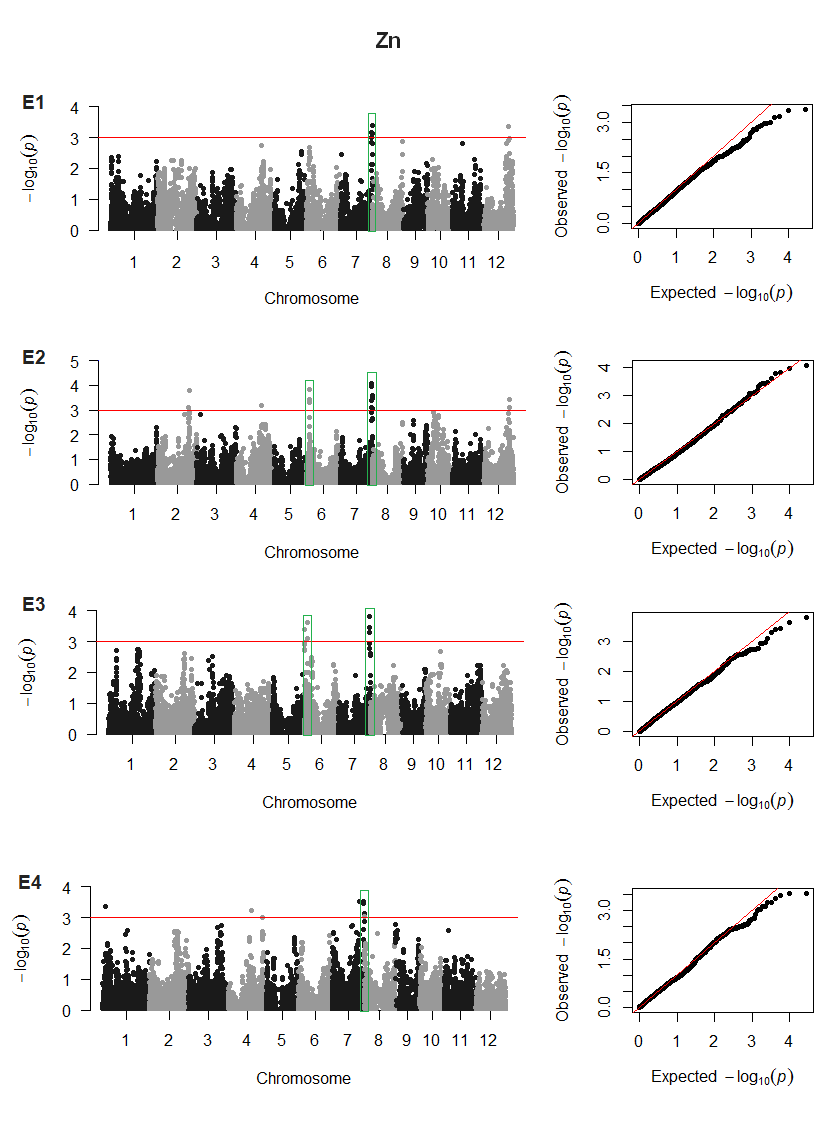

Supplement: FIGURE S12 — Manhattan plots and QQ-plots generated from genome-wide association study of grain zinc concentration observed in the MAGIC Plus population in all environments. Highlighted genomic regions contain SNPs common to at least 2 environments (TIF). [file Image_12.TIF]

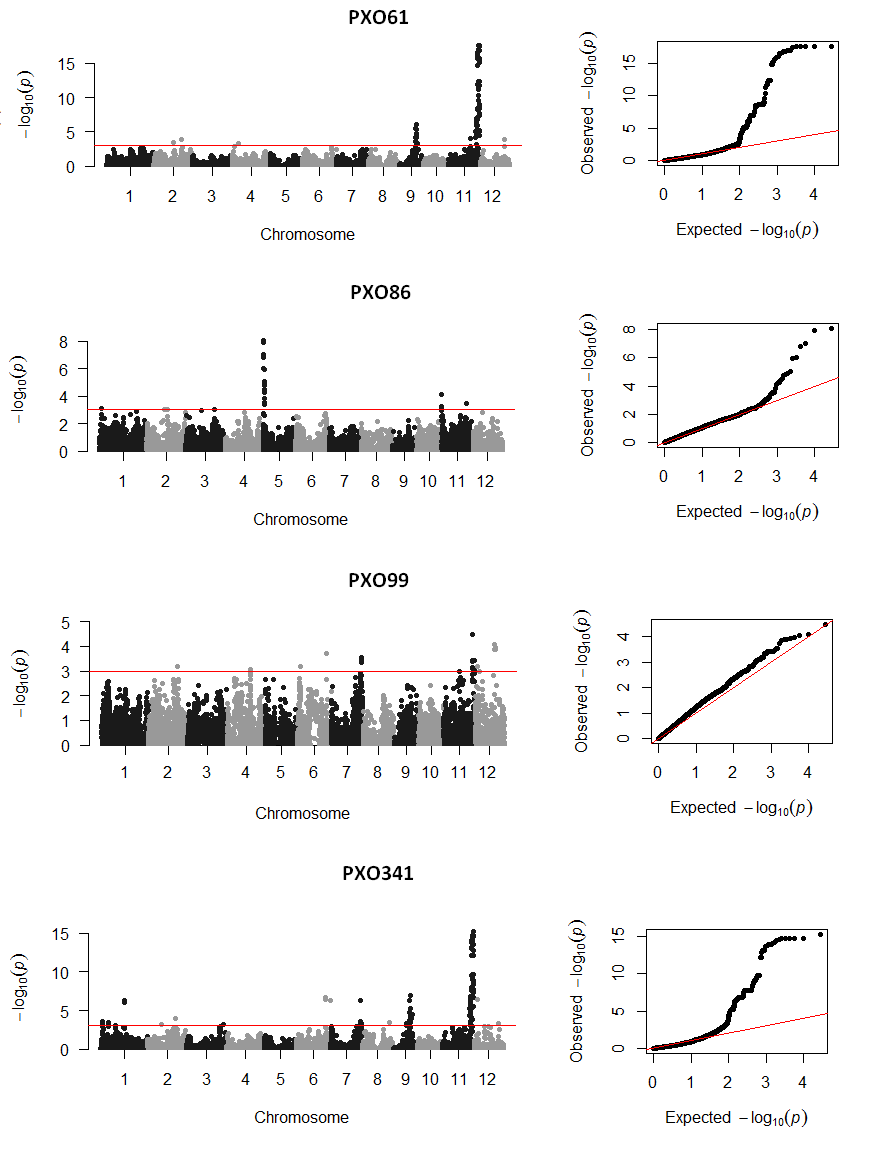

Supplement: FIGURE S13 — Manhattan plots generated from MLM analysis of resistance to four races of bacterial leaf blight disease observed in the MAGIC Plus population (TIF). [file Image_13.TIF]

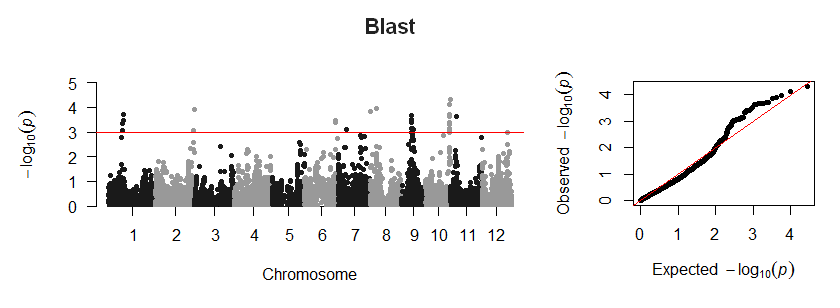

Supplement: FIGURE S14 — Manhattan plots generated from MLM analysis of resistance to blast disease observed in the MAGIC Plus population (TIF). [file Image_14.TIF]
